# Flavonoid Derivatives as New Potent Inhibitors of Cysteine Proteases: An Important Step toward the Design of New Compounds for the Treatment of Leishmaniasis

**DOI:** 10.3390/microorganisms11010225

**Published:** 2023-01-16

**Authors:** Estela Mariana Guimarães Lourenço, Juliana Fortes Di Iório, Fernanda da Silva, Felipe Leonardo Bley Fialho, Melquisedeque Mateus Monteiro, Adilson Beatriz, Renata Trentin Perdomo, Euzébio Guimarães Barbosa, Jean Pierre Oses, Carla Cardozo Pinto de Arruda, Wagner Alves de Souza Júdice, Jamal Rafique, Dênis Pires de Lima

**Affiliations:** 1 Laboratory of Synthesis and Transformation of Organic Molecules-SINTMOL, Institute of Chemistry, Universidade Federal de Mato Grosso do Sul, Av. Senador Filinto Muller, Campo Grande 79074-460, MS, Brazil; 2 Centro Interdisciplinar de Investigação Bioquímica (CIIB), Universidade de Mogi das Cruzes (UMC), Mogi das Cruzes 08780-911, SP, Brazil; 3 Laboratório de Parasitologia Humana, Instituto de Biociências, Universidade Federal de Mato Grosso do Sul, Campo Grande 79070-900, MS, Brazil; 4 Laboratory of Molecular Biology and Cell Culture, School of Pharmaceutical Sciences, Food Technology, and Nutrition, Universidade Federal de Mato Grosso do Sul, Campo Grande 79070-900, MS, Brazil; 5 Laboratório de Química Farmacêutica Computacional, Departamento de Farmácia, Universidade Federal do Rio Grande do Norte, Natal 59012-570, RN, Brazil; 6 Laboratório de Neurociências, Instituto de Biociências, Universidade Federal do Rio Grande, Rio Grande 96203-900, RS, Brazil; 7 Instituto de Química, Universidade Federal de Goiás-UFG, Goiânia 74690-900, GO, Brazil

**Keywords:** synthesis, leishmaniasis, flavonoids, rCPB, molecular modelling

## Abstract

Leishmaniasis is a neglected tropical disease, affecting more than 350 million people globally. However, there is currently no vaccine available against human leishmaniasis, and current treatment is hampered by high cost, side-effects, and painful administration routes. It has become a United Nations goal to end leishmaniasis epidemics by 2030, and multitarget drug strategy emerges as a promising alternative. Among the multitarget compounds, flavonoids are a renowned class of natural products, and a structurally diverse library can be prepared through organic synthesis, which can be tested for biological effectiveness. In this study, we synthesised 17 flavonoid analogues using a scalable, easy-to-reproduce, and inexpensive method. All synthesised compounds presented an impressive inhibition capacity against rCPB2.8, rCPB3, and rH84Y enzymes, which are highly expressed in the amastigote stage, the target form of the parasite. Compounds **3c**, **f12a**, and **f12b** were found to be effective against all isoforms. Furthermore, their intermolecular interactions were also investigated through a molecular modelling study. These compounds were highly potent against the parasite and demonstrated low cytotoxic action against mammalian cells. These results are pioneering, representing an advance in the investigation of the mechanisms behind the antileishmanial action of flavonoid derivatives. Moreover, compounds have been shown to be promising leads for the design of other cysteine protease inhibitors for the treatment of leishmaniasis diseases.

## 1. Introduction

Leishmaniasis comprises a group of vector-borne infectious diseases with a broad clinical spectrum. Classified as a neglected disease by the World Health Organisation (WHO), leishmaniasis affects more than 350 million people worldwide [1,2]. Although it represents a serious public health problem, there is still no vaccine for humans. In addition, the drugs used in therapy are expensive and highly toxic, they cause numerous side-effects, and the routes of administration are painful. Consequently, adherence to treatment is impaired, further strengthening the disease cycle, especially in developing countries [3].

The serious situation involving leishmaniasis resulted in the United Nations (UN) setting a goal in 2020 to combat this group of diseases: sustainable development goal (SDG) 3.3 aims to end the epidemics of several diseases, including neglected tropical diseases (NTDs) by 2030 [4]. This objective has made the discovery and development of new antileishmanial drugs that are more effective, cheaper, easily obtainable, and capable of being administered via alternative routes an emergency. To achieve this goal, different drug discovery approaches need to be used. The multitarget drug strategy has emerged in the last few decades; this approach is based on the complexity of the pathologies and considers that single-target drugs are insufficient to achieve the desired therapeutic effects [5]. Recently, the multitarget drug strategy was reported as a tool to accelerate the discovery of safer, more active, and patient-compliant drugs for the treatment of leishmaniasis [6].

**Figure 1:**
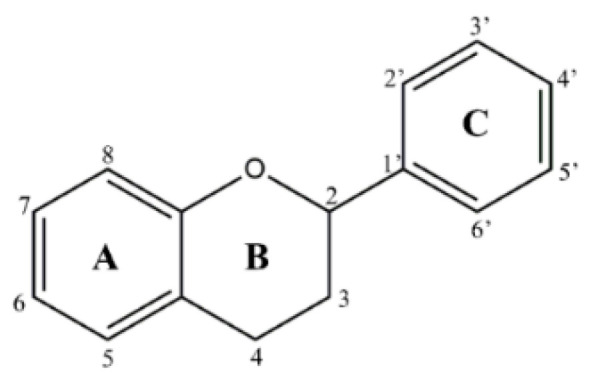
Chemical flavonoid core (C6–C3–C6).

In countries rich in biodiversity, such as Brazil, the use of secondary metabolites from natural sources as new prototypes is a compelling alternative. Flavonoids stand out as they comprise one of the most diverse groups of secondary metabolites, marked by their wide distribution in plants and different therapeutic potentials [7]. These compounds are structurally versatile due to their chemical core and are considered important prototypes for the development of multitarget drugs (Figure 1). It is essential to note that flavonoids have shown in vitro and in vivo antileishmanial activity [8,9]. Flavonols such as quercetin and fisetin inhibit the arginase enzyme, as well as modulate the host’s immune response against the parasite, resulting in low patient toxicity [8]. However, the process of isolating and purifying these compounds is expensive and time-consuming. These methodologies generally require high-cost equipment, result in low yields, and use toxic solvents, reducing the sustainability of the process.

In line with our interest in discovering and developing new sustainable, efficient methodologies for biologically active compounds and a patient-compliant alternative for leishmaniasis treatment [10,11,12,13,14,15,16,17], our research group aimed to yield natural-based bioactive compounds with multitarget properties. To achieve this purpose, we designed a scalable, easy-to-replicate, and inexpensive synthetic route to obtain flavonol and chalcone analogues. The antileishmanial activity was elucidated using in vitro and in silico methods, and the cytotoxicity was measured to determine the selectivity index (SI).

## 2. Results and Discussion

### 2.1. Chemistry

**Figure 2:**
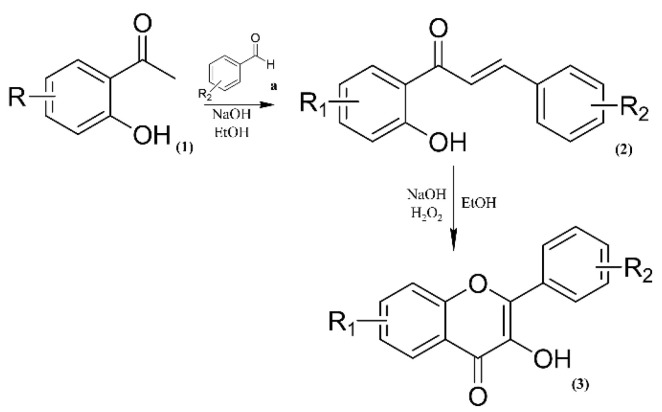
Synthetic route to obtain flavonol-like compounds.

Our retrosynthetic analysis was based on green chemistry principles. Thus, we used mild reaction conditions and inexpensive catalysts and reagents (Figure 2). The chalcone analogue synthesis consists of a Claisen–Schmidt reaction, which is a condensation of aldehydes and carbonyl compounds, leading to α,β-unsaturated ketones in the presence of a base or Lewis acid [18]. In particular, the use of a base as a catalyst provides higher yields of flavonol-like compounds. The intramolecular H-bond of *o*-hydroxyacetophenones leads to an increase in acidity of its α-hydrogen and, in the presence of a base, aids in the generation of a strongly attacking enol anion [19]. Chalcone analogues were used in the synthesis of flavonol-like molecules through the Algar Flynn–Oyamada reaction, in which a chalcone undergoes an oxidative cyclisation under alkaline conditions to form a flavonol [20].

To obtain inexpensive bioactive compounds, we selected low-cost benzaldehydes and acetophenones. The number of synthetic compounds and the structural variations were designed to be statistically relevant for the elucidation of the structure–activity relationship (SAR). Among acetophenones, we also used halogenated *o*-hydroxyacetophenones, as these substituents are reported for their high and selective antimicrobial actions, including antileishmanial activity [21,22]. Previously described aspects of the synthetic process were reviewed to obtain moderate to high yields of the target compounds (Table 1). Our protocol can also be applied to significantly larger reaction mixtures, and this scale-up also gives good yields.

Higher yields of chalcones were achieved when halogenated *o*-hydroxyacetophenones were used. Substituting a halogen atom in the phenolic ring results in an increase in the acidity of the hydroxyl group, which further favours the formation of the enol anion over the formation of unsubstituted *o*-hydroxyacetophenones [23]. Although chalcones exist as trans (*E*) or cis (*Z*) isomers, the *E* isomer is more stable from the thermodynamic perspective [24]. The configuration of the *Z* isomer is unstable as a result of the strong steric effects between the carbonyl group and the A-ring, making the *E* isomer the predominant configuration obtained in our study.

The compounds were unambiguously characterised by NMR spectra. Chalcones **4a–4c** presented most of the ^13^C-NMR signals as doublets due to ^1^*J*, ^2^*J*, and ^3^*J* fluorine (spin ½) coupling with the respective carbons in the aromatic ring. Compared to the starting materials, the chalcones presented CH group signals at 7.30–7.90 ppm, with coupling constants (*J*) of 15.3 Hz, confirming the formation of the *E* isomer. These signals were not observed in the flavonol spectra. Furthermore, compounds **f12a–f13c** presented an OH group signal at 9.60–9.30 ppm, indicating the loss of 2-hydroxy from the starting material (chalcone), classically observed with a larger chemical shift due to the intramolecular H bond with the carbonyl group. The NMR data were compared with the literature, and the structures of the synthesised compounds were confirmed [25,26,27,28,29,30,31,32,33,34,35,36,37]. The spectra are available in the Supplementary Material.

### 2.2. Determination of Inhibitory Potential (IC_50_), Mechanism of Cysteine Protease Inhibition and Evaluation of the Structure–Activity Relationship (SAR)

The discovery and development of new antileishmanial drugs is usually directed through phenotypic or target-based approaches [2]. The target-based strategy is based on previous evidence of the action of compounds on specific pharmacological targets. Driven by advances in molecular biology and the urgency to discover new effective drugs, this approach has been the dominant tool in the last three decades [38]. However, most of the molecules designed by the target-based strategy only demonstrated antipromastigote effects, although amastigote is the target form of the parasite, since this stage occurs in mammalian host cells [39].

*Leishmania* proteases stand out in this context. In particular, cysteine protease B (CPB) expression is elevated in the amastigote stage and plays an important role in the interaction between the parasite and its mammalian host [40]. The results of the inhibition of these proteases indicate their influence on macrophage infection and amastigote survival in host cells, as well as modulating the host’s immune response [41]. The isolated bioflavonoids and their semisynthetic derivatives have demonstrated satisfactory activity against the isoforms rCPB2.8 and rCPB3 [42]; thus, these enzymes represent promising therapeutic targets for our study. 

**Figure 3:**
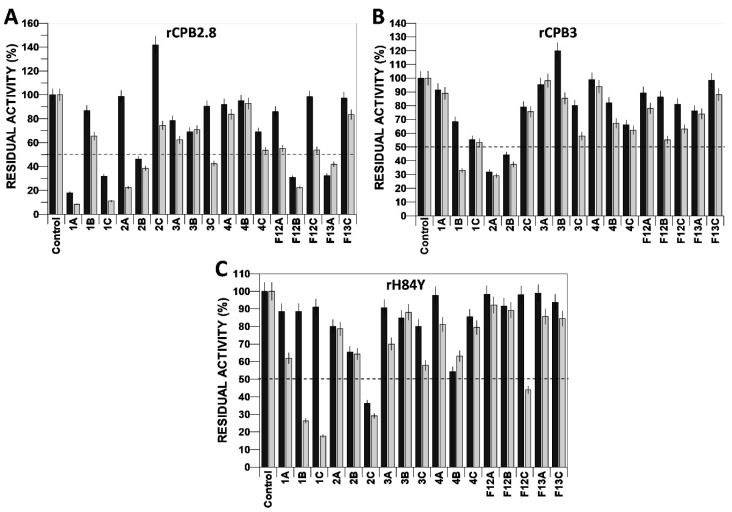
Screening of the action of compounds derived from chalcones and flavonoids on the enzymatic activity of the cysteine protease type B rCPB2.8 (**A**) of *Leishmania mexicana* and its isoforms rCPB3 (**B**) and rH84Y (**C**). Black column: 1 µM compound; grey column: 5 µM compound. Dotted line: delimits residual activity by 50%.

Among the chalcones (**1a–4c**) evaluated on rCPB2.8 (Figure 3A), the compounds **1a** and **1c** stood out as they inhibited 82.02% of enzymatic activity at 1 µM and 91.59% at 5 µM. Compound **f12b**, which reduced the activity by 69.19% and 77.49% at 1 µM and 5 µM, respectively, was the most effective compound in inhibiting rCPB2.8 among the flavonols (**f12a–f13c**). Interestingly, chalcone **2c** increased activity to 141.71% at 1 µM but reduced it by 25.58% at 5 µM.

In the screening of compounds with rCPB3, it was found that chalcone analogues **1b**, **2a**, and **2b** reduced the enzymatic activity by 67.01%, 70.92%, and 62.55%, respectively, at a concentration of 5 µM. Flavonols were unable to inhibit the enzyme by more than 50% at concentrations of 1 µM or 5 µM (Figure 3B). In the inhibition of rH84Y with compounds at 5 µM, it was observed that chalcones **1b**, **1c**, and **2c** reduced activity by 73.61%, 82.29%, and 70.77%, respectively, while the flavonoid **f12c** reduced activity by 56%. Only compound **2c** was able to inhibit more than 50% at 1 µM (Figure 3C).

Since all chalcones and flavonol analogues inhibited the three enzymes to some degree at a concentration of 5 µM, all were subjected to assays to determine their inhibitory potential (IC_50_) against rCPB2.8 and isoforms.

The chalcones with the best inhibitory potential for rCPB2.8 were **1c** (IC_50_ = 2.75 ± 0.18 µM), **2a** (IC_50_ = 2.35 ± 0.19 µM), **3c** (IC_50_ = 3.97 ± 0.08 µM), and **4a** (IC_50_ = 3.35 ± 0.17 µM). The presence of chlorine on carbon C3′ or C4′, fluoride on C3′, or hydroxyl on C6′ seems not to affect the inhibitory capacity of the compounds. However, the presence of the 1,3-dioxolane group, in general, did not favour enzyme inhibition, as observed for **2b**, which was 8.78 times less efficient in inhibiting rCPB2.8 than compound **2a** (Table 2).

In the inhibition of rCPB3, compounds **1b** (IC_50_ = 5.60 ± 0.35 µM), **3a** (IC_50_ = 4.22 ± 0.81 µM), **3c** (IC_50_ = 5.47 ± 0.59 µM), and **4a** (IC_50_ = 4.51 ± 0.19 µM) showed the best inhibitory potential among the chalcone analogues. Compounds bearing a chlorine atom at C3′ (**3a** and **3c**) or a methoxy group at C4 (**3a** and **4a**) had the lowest IC_50_. On the contrary, the compound with the lower IC_50_ over rCPB3 was **4c** (IC_50_ = 37.97 ± 7.24 µM), which has fluoride, hydroxyl, and dimethylamine groups at C3′, C6′, and C4, respectively (Table 2). Chalcones **2b** (IC_50_ = 4.67 ± 0.13 µM), **3c** (IC_50_ = 4.81 ± 0.32 µM), and **4c** (IC_50_ = 4.34 ± 0.43 µM) showed the best inhibitory potentials for rH84Y. Compound **4c** was more efficient in inhibiting rH84Y compared to rCPB2.8 and rCPB3 (Table 2). 

The difference in amino-acid sequence may have affected the inhibitory potential of **4c**. The enzyme rH84Y differs from rCPB3 by a single amino-acid residue [43]. Since **4c** was 8.74 times more potent in inhibiting rH84Y compared to rCPB3, it is possible to infer that the substitution of histidine for tyrosine was the variation that most affected the inhibitory potential of **4c**. 

In the evaluation of the inhibitory capacity of flavonoids against rCPB2.8, the compounds **f12a** (IC_50_ = 4.72 ± 0.38 µM), **f12b** (IC_50_ = 5.23 ± 0.32 µM), and **f13a** (IC_50_ = 1.88 ± 0.07 µM) showed the highest inhibitory potentials. Methoxy added to the C4′ carbon in the **f13a** structure seems to potentiate the enzyme inhibition. On the other hand, the presence of dimethylamine on C4′ in **f13c** disfavoured the inhibition of rCPB2.8 by 7.62-fold. Compounds **f12a** (IC_50_ = 7.71 ± 0.78 µM) and **f12b** (IC_50_ = 7.06 ± 0.63 µM) showed the best IC_50_ values on rCPB3. Again, it was found that the presence of dimethylamine did not favour rCPB3 inhibition, as compounds **f12c** (IC_50_ = 16.67 ± 2.53 µM) and **f13c** (IC_50_ = 29.75 ± 2.08 µM) were the least effective. Regarding the inhibition of rH84Y, the flavonoids **f12a** (IC_50_ = 3.85 ± 0.27 µM) and **f12b** (IC_50_ = 8.85 ± 0.33 µM) presented the best inhibitory potential, and compounds **f12c** and **f13c** had the lowest inhibitory capacity (Table 3). On the basis of these data, it can be observed that the presence of dimethylamine on C4′ carbon negatively impacts the inhibition of the three enzymes.

The high inhibition capacity of all compounds against the three enzyme isoforms confirms their multitarget properties. Studies have shown the formation of larger lesions in BALB/c mice that received amastigotes expressing only CPB2.8 compared to those with amastigotes deficient in all three isoforms [44]. This result confirms that inhibition against the three isoforms is essential when developing a new effective antileishmanial compound based on the inhibition of the CPB enzymes. Among the compounds under study, the chalcone **3c** and flavonols **f12a** and **f12b** were the most effective simultaneously against all enzymes tested, making them good candidates for prototypes. Therefore, these compounds were selected to evaluate the inhibition mechanisms of rCPB2.8, rCPB3, and rH84Y.

By evaluating the inhibition mechanisms of compounds **3c**, **f12a**, and **f12b** on rCPB2.8, we verified the slope vs. [inhibitor] and the intercept vs. [inhibitor] (Figures S1D, S2D, and S3D, Supplementary Materials) of the parabolic profile. This means that two molecules participate in the inhibition mechanism process. Initially, there is binding of the first molecule, which may favour or impair the binding of the second. This molecular behaviour is responsible for respectively establishing the cooperativity as positive or negative. To determine the affinity constants of the compounds, it was necessary to perform the linearisation of the parabolas, obtaining the replots 1/K_Slope_ vs. [inhibitor] (Figures S1E, S2E, and S3E, Supplementary Materials) and 1/K_Intercept_ vs. [inhibitor] (Figures S1F, S2F, and S3F, Supplementary Materials), as well as the factors α, β and γ.

According to the replots 1/K_Slope_ vs. [**3c**] (Figure S1E, Supplementary Materials) and 1/K_Intercept_ vs. [**3c**] (Figure S1F, Supplementary Materials), it was possible to determine the inhibition constants K_i_ = 100 ± 18 μM, αK_i_ = 147 ± 28 μM, βK_i_ = 0.55 ± 0.10 μM, and γK_i_ = 1.15 ± 0.21 μM. The factor α = 1.47 shows that molecule **3c** had a greater preference to bind to the free enzyme. The factors β = 0.0055 and γ = 0.0115 show that binding of the first molecule to form EI or ESI favoured the formation of IEI and IESI by 182- and 87-fold, respectively (Table 4).

The results of the inhibitory mechanism of rCPB2.8 by **f12a** demonstrated a cooperativity inhibition as a function of parabolic replots. Their respective linearisation (Figure S2C–F, Supplementary Materials) allowed the determination of K_i_ = 12.4 ± 1.4 μM and αK_i_ = 10.7 ± 1.1 μM, with α being ~1, considering the standard deviation. The first molecule of **f12a** had the same affinity for binding to the free enzyme E or the ES complex. The values of βK_i_ = 3.65 ± 0.39 μM and γK_i_ = 0.25 ± 0.03 μM defined β = 0.29 and γ = 0.02 (Table 4). Therefore, binding of the first molecule favoured the formation of the IEI and IESI complexes 3.4- and 50-fold, respectively.

In the inhibition of rCPB2.8 by compound **f12b** (Figure S3, Supplementary Materials), the constants K_i_ = 39.2 ± 4.1 μM, αK_i_ = 24.9 ± 2.3 μM, βK_i_ = 1.17 ± 0.12 μM, and γK_i_ = 0.51 ± 0.09 μM were calculated. On the basis of these results, α = 0.634, β = 0.029, and γ = 0.013 were measured (Table 4). The data show that the binding of the first **f12a** facilitated the formation of IEI 34-fold and the formation of IESI 77-fold. The established mechanism is non-competitive, with positive cooperativity.

Parabolic replots were observed for the inhibition of rCPB3 by compound **3c** (Figure S4B,C, Supplementary Materials). However, compounds **f12a** and **f12b** (Figures S5 and S6, Supplementary Materials) demonstrated linear slope and linear intercept replots (Figures S5B,C and S6B,C, Supplementary Materials). The inhibition constants determined for **f12a** were K_i_ = 7.47 ± 0.64 μM and αK_i_ = 7.50 ± 0.66 μM. Compound **f12b** presented K_i_ = 13.7 ± 1.4 μM and αK_i_ = 13.7 ± 1.2 μM, and both α values were 1, which means that the compounds bind with the same affinity to the free enzyme E or to the ES complex. The **3c** inhibition constants were K_i_ = 15.4 ± 3.0 μM, αK_i_ = 141 ± 27 μM, βK_i_ = 2.21 ± 0.16 μM (*β* = 0.14), and γK_i_ = 0.77 ± 0.14 μM (*γ* = 0.05) (Table 4). The α value (α = 9.15) shows that **3c** preferentially binds to the free enzyme. With the binding of the compound to the ES complex that forms ESI defined by the γ factor, the formation of the quaternary IESI complex will be favoured 20-fold. On the other hand, the formation of the EI complex by binding of the first molecule **3c** favours the formation of IEI sevenfold. Therefore, **3c** presented a non-competitive inhibition mechanism with positive cooperativity, while **f12a** and **f12b** presented a simple linear non-competitive inhibition mechanism.

Compounds **3c**, **f12a**, and **f12b** showed rH84Y inhibition profiles similar to those observed in the inhibition of rCPB3 (Figures S7–S9, Supplementary Materials). The affinity constants determined for **3c** were K_i_ = 29.4 ± 4.7 μM, αK_i_ = 127 ± 20 μM, βK_i_ = 1.45 ± 0.27 μM, and γK_i_ = 1.06 ± 0.18 μM (Figure S7E,F, Supplementary Materials). The calculated factors were α = 4.31, β = 0.049, and γ = 0.036 (Table 4). Thus, the binding of the first **3c** molecule to the free enzyme favoured the formation of IEI 20-fold, and the formation of IESI was favoured 28-fold. The effects of compounds **f12a** and **f12b** showed slope and linear intercept replots with values of inhibition constants K_i_ = 11.2 ± 0.8 μM and αK_i_ = 11.2 ± 0.5 μM for **f12a**, and Ki = 6.08 ± 0.37 μM and αK_i_ = 6.06 ± 0.24 μM for **f12b**, with α ≈ 1 in both cases (Figures S8C,D and S9C,D, Supplementary Materials). Therefore, **3c** presented a non-competitive inhibition mechanism with positive cooperativity, and **f12a** and **f12b** presented a simple linear non-competitive inhibition mechanism.

### 2.3. Molecular Modelling Study

The results of inhibitory capacity and the mechanism of action against these isoforms by the obtained chalcone and flavonol analogues are unprecedented. However, previous work in the literature may shed light on understanding the potential binding mode of the compounds at the active site of the CPB isoforms. *Leishmania mexicana* type B cysteine proteases are L-like cathepsins. The inhibition activity of these enzymes by chalcones is already known. Studies by Raghav and Kaur found that the catalytic CYS 29 thiolate of cathepsin L was able to attack the nucleophilic sites of chalcones [45]. In other recent work, chalcones demonstrated in vitro antileishmanial activity on the amastigote and promastigote forms of *L. infantum*. The suggested mechanism of action was the inhibition of pro-cathepsin L-like by the formation of hydrogen bonds between the amino acid TRP 151 of the active site and the carboxyl group of the chalcones [46]. Isolated flavonoids were also able to inhibit cathepsins L and B in previous studies [47].

**Figure 4:**
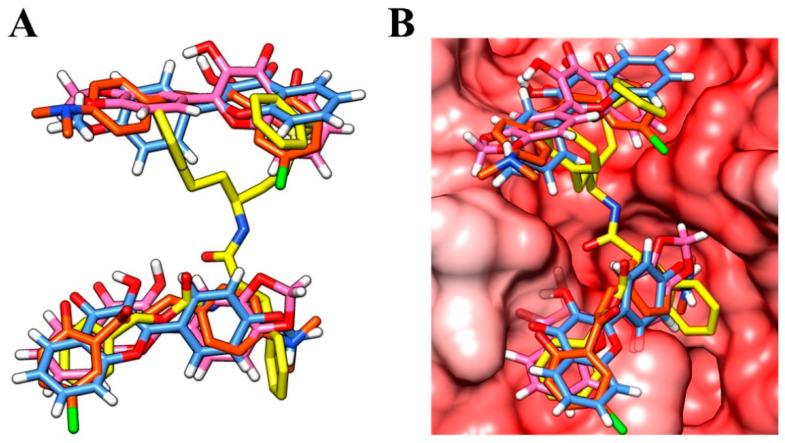
(**A**) Overlay of the vinyl sulphone analogue (yellow), compound **3c** (orange), **f12a** (blue), and **f12b** (pink); (**B**) the compounds overlaid at the binding site of the enzyme.

Other classes of small molecules have also been described as inhibitors that target multiple cathepsin L-like cysteine proteases, some with overlapping antiparasitic activity [40]. Among them, vinyl sulphones have been shown to be highly potent and selective inhibitors of cathepsins L and B and are also considered antiparasitic prototypes [48,49]. Interestingly, a remarkable 3D similarity is demonstrated by the structural overlap of a crystallographic analogue of vinyl sulphone and compounds **3c**, **f12b,** and **f12c**; as a result, there is a compatible binding mode at the active site of the enzyme (Figure 4A,B, respectively). In particular, the overlap with the vinyl sulphone was useful in understanding non-competitive inhibition with a positive cooperativity mechanism. Therefore, the crystal structure of a papain-like cysteine protease bound with the vinyl sulphone derivative [50] was used as a template in the homology modelling study.

The isoforms rCPB2.8, rCPB3, and rH84Y have a small number of modifications in their active sites (Table S1, Supplementary Material). However, the few amino-acid variations between these isoenzymes are important in modifying substrate specificities [43]. This change may have modified either the catalytic or allosteric site, even at a distance. This type of event was observed in substitutions distant from the active site that affect the catalytic activity of CheZ and the binding of CheYp, with possible propagation of structural or dynamic disturbance [51].

Interestingly, even with the amino-acid residue variations, chalcone **3c** showed the same mechanism of action on the three isoforms. To investigate its possible binding mode at the active site of the enzymes, the energy values of each pose pointed by 3D overlapping at rCPB2.8 was calculated after geometry optimisation (Table S2, Supplementary Materials). The position with the lower potential energy was also used to investigate the intermolecular interactions at the active site of rCPB3 and rH84Y. Despite having the same mechanism of action, the calculated binding free energy values of compound **3c** at the catalytic site of the isoforms were remarkable different (Table 5). Particularly, the chalcone had the most promising binding free energy result on rCPB3 (−24.70 kcal·mol^−1^). These values reinforce the hypothesis of a more favourable interaction in a molecular scenario between compound **3c** and this isoform and corroborates with the in vitro results. 

On the contrary, the variation of amino-acid residues resulted in a different mechanism of action for flavonols **f12a** and **f12b** between the three isoforms. This change is due to the difference in the negative charge distribution of these residues, which necessarily results in significant changes in the electrostatic potential on the surface of the isoenzymes, in addition to providing the parasite with a series of hydrolytic activities [43,52]. Therefore, the change in the electrostatic potential on the surface of the isoenzymes promoted changes in the inhibition mechanisms, as well as differences in the affinity constants.

Following the simple linear non-competitive inhibition mechanism, the potential energy of the different positions of **f12a** at the binding site of rCPB3 was promising (Table S3, Supplementary Materials). The binding pose with the lower potential energy was used to investigate the intermolecular interactions of the flavonol analogues at the active site of rCPB3 and rH84Y, since the mechanism of action was the same at these two isoforms. As also occurred with compound **3c**, the calculated binding free energy values of **f12a** at the binding site of the isoforms were notably different, with the lowest value for rCPB3 (−5.65 kcal·mol^−1^). This result corroborates the in vitro assays that demonstrate a higher affinity constant for this isoform (Table 5). Despite the structural similarity, compound **f12b** had a lower binding free energy at the simulations of the active site of rH84Y (Table 5). The calculated energy values also demonstrated a great difference among the three isoforms (Table 5), which corroborates the considerably lower affinity constant for rCPB2.8. 

**Figure 5:**
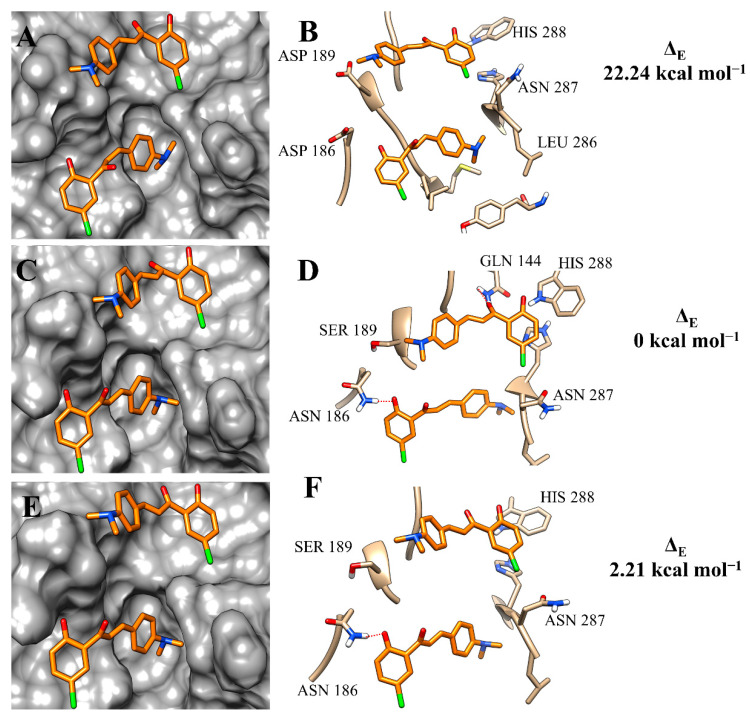
Binding mode positions of chalcone **3c** at the active site of rCPB2.8 (**A**,**B**), rCPB3 (**C**,**D**), and rH84Y (**E**,**F**). The hydrogen bonds are represented by a red dashed line.

Analysing the output results of the simulations of compound **3c**, the variations of amino-acid residues at the binding site of the isoforms resulted in a similar occupation of the binding pockets, but noticeable differences in the intermolecular interactions (Figure 5A,C,E). At the binding site of rCPB2.8, **3c** mainly made hydrophobic interactions (Figure 5B). However, the substitution of ASP 186 to ASN 186 resulted in the formation of a hydrogen bond, which was also observed with GLY 144 (Figure 5D). This strong intermolecular interaction was conserved at the active site of rH84Y; however, the hydrogen bond with GLY 144 was not observed at the catalytic site of this isoform (Figure 5F). The difference in intermolecular interactions, added to the binding-free energy values, corroborates the affinity constants obtained by in vitro assays. 

**Figure 6:**
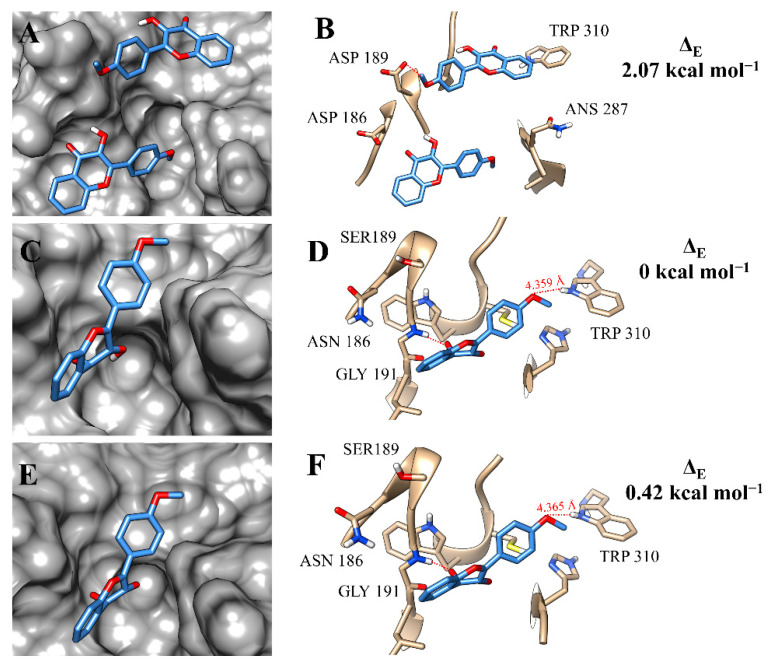
Binding mode positions of flavonol **f12a** at the active site of rCPB2.8 (**A**,**B**), rCPB3 (**C**,**D**), and rH84Y (**E**,**F**). The hydrogen bonds are represented by a red dashed line.

At the active site of rCPB2.8, the molecules of flavonol **f12a** had a great occupation of the binding pockets and made hydrogen bonds with the amino-acid residue ASP 189 (Figure 6A,B). The number of hydrogen bonds increased at the binding site of rCPB3, since the compound made interactions with TRP 310 and GLY 191 (Figure 6D). Previous studies have already discussed the interaction between flavonoid derivatives and the GLY amino-acid residue of the cathepsin L catalytic site, as well as its importance in stabilising the active compound at the binding site of the enzyme [45]. The occupation of **f12a** at the binding pockets of rCPB3 and rH84Y was very similar (Figure 6C,E). This resemblance was reflected by the interactions with the amino-acid residues of the two isoforms (Figure 6F).

**Figure 7:**
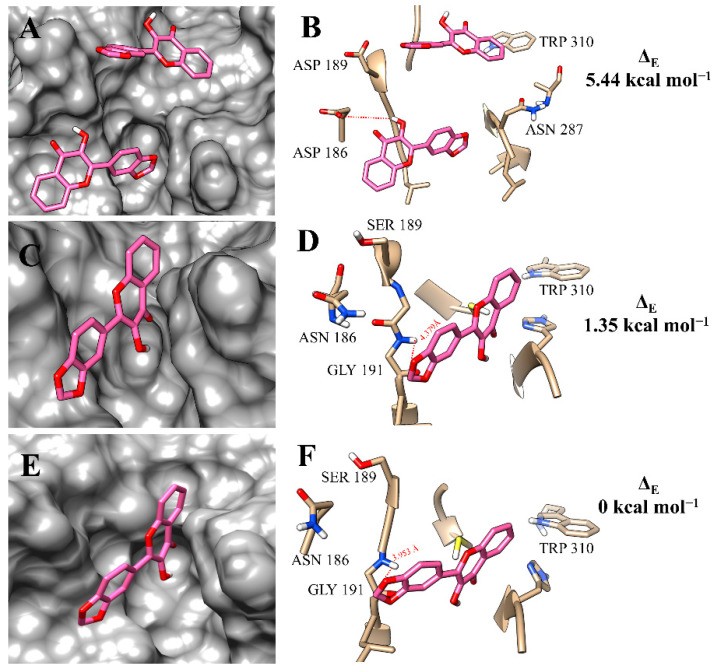
Binding mode positions of flavonol **f12b** at the active site of rCPB2.8 (**A**,**B**), rCPB3 (**C**,**D**), and rH84Y (**E**,**F**). The hydrogen bonds are represented by a red dashed line.

The binding poses of **f12b** with the rCPB isoforms were very similar to those found for **f12a** and highly resembled the binding poses of each isoform, even by the simple linear non-competitive inhibition mechanism (Figure 7A,C,E). At the active site of rH84Y, the proximity with the amino-acid residue GLY 191 (3.953 Å) may be related to a better interaction with this isoform (Figure 7F).

### 2.4. Antipromastigote Assay and Cytotoxicity Elucidation

Although flavonoid derivatives have demonstrated promising enzyme-inhibitory potential, the development of a drug candidate for leishmaniasis treatment depends on several pharmacological aspects. Among them, the cytotoxicity of compounds is crucial for the discovery of a new antileishmanial prototype, since high toxicity still represents a serious limitation of the drugs used in current therapy [53]. The compounds need to be highly active against the *Leishmania* parasite and provide safety to host cells. This pharmacological characteristic is measured by SI, defined as the ratio of the 50% cytotoxic concentration of mammalian cells (GL_50_) to the half-maximum inhibitory concentration on parasites (IC_50_). 

However, the determination of IC_50_ by in vitro screening tests is a challenge with the *Leishmania* parasite itself. The *Leishmania* lifecycle requires the presence of a sand fly vector and a mammalian host that causes the existence of two distinct morphological forms (promastigote and amastigote). Although the amastigote form is found in host cells and is considered the target form of the parasite, determination of the IC_50_ consists of a time-consuming and laborious procedure and is not suitable for a large-scale screening method [54]. In general, exploratory screening methods designed to accelerate the testing of many compounds are performed on the promastigote form [54,55]. Therefore, to determine the SI of all flavonoid derivatives, we used the half-maximum inhibitory concentration on promastigote forms.

All 17 flavonoid derivatives had moderate to low solubility in water. This physical property represented an obstacle in determining the IC_50_ of the compounds, as the test occurred in an aqueous medium. Consequently, the higher concentration tested (10 µg/mL) was lower than that generally described in the literature (50 µg/mL) [56]. Compounds with IC_50_ greater than 10 µg/mL were considered nonactive. However, since even the higher concentration tested was lower than that reported for compounds considered active against the parasite, these molecules may not be excluded as potential antileishmanial prototypes.

Among the tested molecules, the chalcone analogues stood out. Compounds **2a**, **3a**, and **4c** were more active than the standard drug pentamidine (IC_50_ = 0.71, 0.60, and 0.50 µM, respectively). Meanwhile, **f12c** had the highest potency of the flavonol derivatives against the parasite (IC_50_ = 0.73 µM). The results indicated that the substitution of a chloride atom in the phenolic ring of the chalcone and flavonol derivatives increased the activity against the *Leishmania* promastigote form (Table 6).

Compounds **3a** and **4c**, the most active against the promastigote form, were also noncytotoxic against mammal cells (Figure S10, Supplementary Materials), with an optimal selective index (SI > 1752.48 and 1443.10, respectively) when compared to first-line drugs such as pentamidine and amphotericin B [56] (Table 6). It is important to emphasise that the compounds tested all demonstrated low cytotoxicity to mammal cells, resulting in high SI values. 

The lipophilicity (logP) and water solubility (logS) properties of the flavonoid derivatives were also measured by in silico analysis. The importance of these chemical characteristics was first discussed by Lipinski et al. through the publication of the rule of five. In this study, among the physicochemical characteristics of a set of standard drugs, clogP ≤ 5 was postulated as being necessary for an ideal prototype [57]. Later, the development of an ADME in silico tool, based on the analysis of more than 2000 standard drugs, indicated that more than 80% of the drugs on the market have a (estimated) logS value greater than −4 [58]. All 17 flavonoid derivatives had logS values close to −4 and logP ≤ 5, following the characteristics postulated by the rule of five. Although further studies against amastigote form need to be carried out, the ADME results, added to the biological potential, indicate that all synthetic flavonoids are druglike and can be considered promising prototypes for the treatment of Leishmaniasis disease.

**Table 6:** Molecular LogP, LogS, in vitro antileishmanial activity and cytotoxicity of flavonoid analogues.

Entry	Compound	LogP_o/w_ ^1^	LogS ^2^	Promastigote IC_50_ (µM) ^3^	NIH/3T3 Cells GL_50_ (µM) ^5^	SI ^6^
1	**1a**	3.97	−4.59	n.a ^4^	294.13	n.d ^7^
2	**1b**	3.87	−5.03	n.a ^4^	811.27	n.d ^7^
3	**1c**	4.04	−4.91	1.14	>874.83	>767.39
4	**2a**	3.10	−4.05	0.71	940.14	1324
5	**2b**	2.88	−4.08	n.a ^4^	910.58	n.d ^7^
6	**2c**	3.17	−4.20	n.a ^4^	>935.21	n.d ^7^
7	**3a**	3.64	−4.64	0.60	>865.86	>1443.10
8	**3b**	3.46	−4.67	0.96	59.13	61.59
9	**3c**	3.65	−4.79	n.a ^4^	>828.44	n.d ^7^
10	**4a**	3.42	−4.20	n.a ^4^	901.09	n.d ^7^
11	**4b**	3.24	−4.23	1.95	>873.36	n.d ^7^
12	**4c**	3.42	−4.36	0.50	>876.24	>1752.48
13	**f12a**	2.83	−4.09	n.a ^4^	>931.93	n.d ^7^
14	**f12b**	2.68	−4.11	n.a ^4^	>885.74	n.d ^7^
15	**f12c**	2.85	−4.23	0.73	759.13	1039.90
16	**f13a**	3.27	−4.44	1.08	>825.87	>764.70
17	**f13c**	3.29	−4.59	n.a ^4^	>791.76	n.d ^7^
Doxorubicin		---	---	---	0.05	n.d ^7^
Pentamidine		---	---	0.80	---	n.d ^7^
Amphotericin B		---	---	0.10	---	n.d ^7^

^1^ LogP, octanol/water partition coefficient measured by SwissADME [59]; ^2^ LogS expressed as log (g/100 g water) measured by SwissADME [59], ^3^ IC_50_, half-maximum inhibitory concentration on promastigote forms; ^4^ n.a, not active (IC_50_ > 10 µg/mL); ^5^ GL_50_, concentration that inhibited cell growth by 50%; ^6^ SI (selectivity index), IC_50_ in mammalian cells/IC_50_ in extracellular promastigotes; ^7^ n.d, not determined. The data are representative of three independent experiments.

## 3. Conclusions

Our synthetic protocols confirmed that the methods are versatile, scalable, easy to reproduce, and inexpensive for obtaining high yields of flavonoid derivatives. The compounds demonstrated the multitarget properties intended by our study to inhibit all tested rCPB isoforms of *L. mexicana*. All chalcones and flavonol analogues inhibited the three enzymes to some degree at a concentration of 5 µM and were subjected to assays to determine their inhibitory potential IC_50_ against rCPB2.8 and isoforms. Regarding the activity of chalcones, the presence of chlorine attached on carbon C3′ or C4′, fluoride on C3′, or hydroxyl on the C6′ seems not to affect the inhibitory capacity of the compounds toward rCPB2.8. However, the presence of the 1,3-dioxolane group, in general, did not favour this enzyme inhibition. Interestingly, the chalcones bearing a chlorine atom at C3′ (**3a** and **3c**) or a methoxy group at C4 (**3a** and **4a**) had the lowest IC_50_ on rCPB3, on the rH84Y isoform and compound **4c** had the lowest IC_50_ on the rH84Y isoform. In the evaluation of the inhibitory capacity of flavonoids, it was observed that the presence of dimethylamine on the C4′ carbon negatively impacted the inhibition of the three enzymes. Among the flavonoid analogues, compounds **3c**, **f12a**, and **f12b** stood out for being effective against all isoforms simultaneously. Interestingly, the in vitro study of the mechanism of cysteine protease inhibition showed that small variations of amino-acid residues between the rCPB isoforms were able to change the mechanism of action and binding mode position of the compounds. These findings were confirmed by the in silico investigation that demonstrated the formation of strong intermolecular interactions between the compounds and the active site of each enzyme. The compounds were highly potent and demonstrated low cytotoxic action against mammalian cells, proving that the tested molecules are extremely selective. In addition, the calculated logP and logS values proved that the compounds have ADME properties compatible with those observed in standard drugs. The antileishmanial activity of all flavonoid analogues needs to be elucidated against amastigote form in future studies. However, our results show important progress in the investigation of the antileishmanial action of synthetic flavonoid derivatives and reinforce their potential as prototypes for the design of other cysteine protease inhibitors for the treatment of leishmaniasis.

## 4. Materials and Methods

### 4.1. Chemistry

All reagents were purchased from Sigma-Aldrich^®^ and were analytical grade, used without further purification. Reactions were monitored by TLC using Merck 60 F254 precoated silica plates, and spot visualisation was achieved with UV light (254–360 nm), molybdophosphoric acid (10% *w/v*), and a solution of sulphur vanillin (0.5 g vanillin in 100 mL of sulphuric acid/methanol (40:10)). All products were purified by recrystallisation from ethanol (EtOH). The solvents used in the reactions and recrystallisation were purified and dried according to procedures found in the literature [60]. A mixture of hexane and ethyl acetate was used in a 1:2 (*v/v*) proportion as the mobile phase to measure the retention factor (R*_f_*) values of all purified compounds. All melting points were determined using a Quimis^®^ of Brazil model Q340S instrument. The ^1^H- and ^13^C-NMR spectra were recorded on a Bruker Avance DPX-300 or Bruker Ascend 500 spectrometer. Chemical shifts are reported as δ values (ppm) referenced to the residual solvent (CDCl_3_ at δ 7.24 ppm, DMSO-*d_6_* at δ 2.50 ppm). Peak multiplicities are abbreviated as follows: s (singlet); d (doublet); dd (doublet of doublets); tp (triplet of doublets); t (triplet); dt (doublet of triplets); m (multiplet). The coupling constants (*J*) are quoted in hertz and recorded at the nearest 0.1 Hz. 

### 4.2. General Procedure for the Synthesis of Chalcone-Like Compounds by the Claisen–Schmidt Reaction (**1a**–**4c**)

The synthesis procedure followed the Claisen–Schmidt reaction methodology described in the literature, with modifications [33]. An aqueous solution of NaOH (3 M, 1.6 mL) was added to a solution of aromatic ketone (1 mmol) in EtOH. An ethanolic solution of substituted benzaldehyde was added dropwise to the reaction mixture. The mixture was stirred at room temperature for 24 h and then cooled. The reaction mixture was acidified with concentrated HCl (37%) to pH = 2 in an ice bath and under vigorous stirring. The precipitate formed was filtered, washed with cold water, and purified by recrystallisation from ethanol. 

### 4.3. General Procedure for the Synthesis of Flavonol-Like Compounds by the Algar Flynn–Oyamada Reaction (**f12a**–**f13c**)

In a round-bottom flask, an aqueous solution of NaOH (1 M, 2 mL) was added to 1 mmol of chalcone in EtOH (5 mL). The solution was cooled until an ice-cold suspension was formed. An aqueous solution of H_2_O_2_ (35%, 250 µL) was added to the ice-cold suspension; the mixture was allowed to warm to room temperature and stirred for 1–2 h. Distilled water (3 mL) was then added. The reaction mixture was acidified with concentrated HCl (37%) to pH = 2 in an ice bath and under vigorous stirring. The precipitate formed was filtered, washed with cold water, and purified by recrystallisation from ethanol.

(*E*)-1-(4-chlorophenyl)-3-(4-methoxyphenyl)prop-2-en-1-one (**1a**): pale yellow crystal. Yield 67.11%. Mp 118–120 °C. R*_f_* = 0.535. ^1^H-NMR (CDCl_3_, 300 MHz) δ ppm 7.96 (2H, d, *J* = 8.6, ArH), 7.81 (1H, d, *J* = 15.6 Hz, C=CH), 7.61 (2H, d, *J* = 8.7, ArH), 7.47 (2H, d, *J* = 8.6, ArH), 7.38 (1H, d, *J* = 15.6 Hz, C=CH), 6.95 (2H, d, *J* = 8.7, ArH), 3.85 (3H, s, –OCH_3_); ^13^C-NMR (CDCl_3_, 75 MHz) δ ppm 189.29, 161.97, 145.32, 139.05, 136.92, 130.45, 129.94, 128.98, 127.54, 119.27, 114.58, 55.54.
(*E*)-3-(benzo[d][1,3]dioxol-5-yl)-1-(4-chlorophenyl)prop-2-en-1-one (**1b**): pale yellow crystal. Yield 86.19%. Mp 126–130 °C. R*_f_* = 0.56. ^1^H-NMR (CDCl_3_, 300 MHz) δ ppm 7.96 (2H, d, *J* = 8.5, ArH), 7.76 (1H, d, *J* = 15.6 Hz, C=CH), 7.48 (2H, d, *J* = 8.5, ArH), 7.33 (1H, d, *J* = 15.6 Hz, C=CH), 7.15 (1H, d, *J* = 1.6 Hz, ArH), 7.13 (1H, dd, *J* = 8.1, ArH), 6.85 (1H, dd, *J* = 8.1, ArH), 6.03 (2H, s, –OCH_2_O); ^13^C-NMR (CDCl_3_, 75 MHz) δ ppm 188.69, 149.80, 148.16, 144.88, 138.74, 136.38, 129.52, 128.87, 125.13, 119.16, 108.41, 106.35, 101.40.
(E)-1-(4-chlorophenyl)-3-(4-(dimethylamino)phenyl)prop-2-en-1-one (**1c**): yellow amorphous powder. Yield 73.26%. Mp 138–140 °C. R*_f_* = 0.40. ^1^H-NMR (CDCl_3_, 300 MHz) δ ppm 7.96 (2H, d, *J* = 8.6, ArH), 7.82 (1H, d, *J* = 15.5 Hz, C=CH), 7.55 (2H, d, *J* = 8.9, ArH), 7.46 (2H, d, *J* = 8.6, ArH), 7.30 (1H, d, *J* = 15.5 Hz, C=CH), 6.70 (2H, d, *J* = 8.9, ArH), 3,04 (6H, s, –N(CH_3_)_2_); ^13^C-NMR (CDCl_3_, 75 MHz) δ ppm 189.94, 151.86, 146.08, 138.12, 137.10, 130.25, 129.43, 128.42, 122.13, 115.90, 111.50, 39.81.
(E)-1-(2-hydroxyphenyl)-3-(4-methoxyphenyl)prop-2-en-1-one (**2a**): yellow amorphous powder. Yield 76.53%. Mp 90–94 °C. R*_f_* = 0.60. ^1^H-NMR (CDCl_3_, 300 MHz) δ ppm 12.96 (1H, s, OH), 7.92 (1H, dd, *J* = 2.3, 8.6 Hz, ArH), 7.91(1H, d, *J* = 15.5 Hz, C=CH), 7.62 (2H, d, *J* = 8.6 Hz, ArH), 7.55 (1H, d, *J* = 15.5 Hz, C=CH), 7.50–7.45 (1H, m, ArH), 7.02 (1H, d, *J* = 8.4 Hz, ArH), 6.95 (2H, d, *J* = 8.4 Hz, ArH), 7.00–6.90 (1H, m, ArH), 3,86 (3H, s, –OCH_3_); ^13^C-NMR (CDCl_3_, 75 MHz) δ ppm 193.35, 163.23, 161.72, 145.04, 135.82, 130.24, 129.22, 127.02, 119.80, 118.44, 118.25, 117.25, 114.20, 55.12.
(*E*)-3-(benzo[d][1,3]dioxol-5-yl)-1-(2-hydroxyphenyl)prop-2-en-1-one (**2b**): yellow amorphous powder. Yield 71.43%. Mp 138–140 °C. R*_f_* = 0.64. ^1^H-NMR (CDCl_3_, 300 MHz) δ ppm 12.89 (1H, s, OH), 7.90 (1H, 1.7, 8.1 Hz, ArH), 7.86 (1H, d, *J* = 15.5 Hz, ArH), 7.50 (1H, ddd, *J* = 1.7, 7.9, 8.1 Hz, ArH), 7.50 (1H, d, *J* = 15.5 Hz, ArH), 7.17 (1H, d, *J* = 1.7 Hz, ArH), 7.15 (1H, dd, *J* = 1.7, 8.1 Hz, ArH), 7.03 (1H, d, *J* = 8.1 Hz, ArH), 6.92 (1H, t, *J* = 7.2, 7.5, Hz, ArH), 6.86 (1H, d, *J* = 7.9 Hz, ArH), 6.03 (2H, s, –OCH_2_O); ^13^C-NMR (CDCl_3_, 75 MHz) δ ppm 193.23, 163.25, 150.0, 148.20, 145.02, 135.91, 129.21, 128.77, 125.44, 119.76, 118.48, 118.29, 117.69, 108.44, 106.44, 101.45.
(E)-3-(4-(dimethylamino)phenyl)-1-(2-hydroxyphenyl)prop-2-en-1-one (**2c**): purple crystals. Yield 82.34%. Mp 178–181 °C. R*_f_* = 0.71. ^1^H-NMR (CDCl_3_, 300 MHz) δ ppm 13.23 (1H, s, OH), 7.93 (1H, d, *J* = 15.4, C=CH), 7.90 (1H, d, *J* = 5.2 Hz, ArH), 7.57 (2H, d, *J* = 8.9 Hz, ArH), 7.48 (1H, d, *J* = 5.2 Hz, ArH), 7.46 (1H, d, *J* = 15.4, C=CH), 7.01 (1H, d, *J* = 7.9 Hz, ArH), 6.94 (1H, t, *J* = 7.7 Hz, ArH), 6.69 (2H, d, *J* = 8.9 Hz, ArH), 3.04 (6H, s, –N(CH_3_)_2_); ^13^C-NMR (CDCl_3_, 75 MHz) δ ppm 193.18, 163.16, 152.02, 146.25, 135.32, 130.55, 129.06, 121.97, 120.08, 118.26, 118.13, 113.90, 111.48, 39.76.
(E)-1-(5-chloro-2-hydroxyphenyl)-3-(4-methoxyphenyl)prop-2-en-1-one (**3a**): yellow amorphous powder. Yield 90.40%. Mp 108–109 °C. R*_f_* = 0.79. ^1^H-NMR (CDCl_3_, 500 MHz) δ ppm 12.87 (1H, s, OH), 7.93 (1H, d, *J* = 15.5, C=CH), 7.85 (1H, s, ArH), 7.65 (2H, d, *J* = 8.1 Hz, ArH), 7.45–7.41 (2H, d, *J* = 15.5, C=CH, ArH), 6.98 (3H, m, ArH), 3.87 (3H, s, –OCH_3_); ^13^C-NMR (CDCl_3_, 125 MHz) δ ppm 192.53, 162.17, 161.87, 146.30, 135.77, 130.69, 128.57, 126.92, 123.27, 120.57, 120.03, 116.66, 114.45, 55.35.
(*E*)-3-(benzo[d][1,3]dioxol-5-yl)-1-(5-chloro-2-hydroxyphenyl)prop-2-en-1-one (**3b**): yellow amorphous powder. Yield 86.70%. Mp 142–146. R*_f_* = 0.72. ^1^H-NMR (CDCl_3_, 500 MHz) δ ppm 12.80 (1H, s, OH), 7.87 (1H, d, *J* = 15.3 Hz, C=CH), 7.83 (1H, d, *J* = 2.5 Hz, ArH), 7.43 (1H, d, *J* = 8.9, 2.5 Hz, ArH), 7.39 (1H, d, *J* = 15.3 Hz, C=CH), 7.19 (1H, d, *J* = 1.7 Hz, ArH), 6.98 (1H, d, *J* = 8.9 Hz, ArH), 6.87 (1H, *J* = 8.1 Hz, ArH), 6.05 (2H, s, –OCH_2_O); ^13^C-NMR (CDCl_3_, 125 MHz) δ ppm 192.43, 161.88, 150.50, 148.44, 146.27, 135.86, 128.65, 128.54, 126.02, 123.33, 120.52, 120.06, 117.08, 108.67, 106.71, 101.72.
(E)-1-(5-chloro-2-hydroxyphenyl)-3-(4-(dimethylamino)phenyl)prop-2-en-1-one (**3c**): purple crystals. Yield 81.74%. Mp 161–163 °C. R*_f_* = 0.68. ^1^H-NMR (CDCl_3_, 300 MHz) δ ppm 13.16 (1H, s, OH), 7.95 (1H, d, *J* = 15.1 Hz, C=CH), 7.85 (1H, d, *J* = 2.1 Hz, ArH), 7.58 (2H, d, *J* = 8.7 Hz, ArH), 7.41 (1H, dd, *J* = 8.8, 2.2 Hz, ArH), 7.35 (1H, d, *J* = 15.1 Hz, C=CH), 6.98 (1H, d, *J* = 8.8 Hz, ArH), 6.70 (2H, d, *J* = 8.7 Hz, ArH), 3.06 (6H, s, –OCH_3_); ^13^C-NMR (CDCl_3_, 75 MHz) δ ppm 192.05, 161.65, 152.25, 147.29, 135.00, 130.87, 128.75, 122.86, 121.75, 120.76, 119.70, 113.08, 111.48, 39.76.
(E)-1-(5-fluoro-2-hydroxyphenyl)-3-(4-methoxyphenyl)prop-2-en-1-one (**4a**): yellow amorphous powder. Yield 84.32%. Mp 124–125 °C. R*_f_* = 0.68. ^1^H-NMR (CDCl_3_, 300 MHz) δ ppm 12.63 (1H, s, OH), 7.95 (1H, d, *J* = 15.3 Hz, C=CH), 7.65 (2H, *J* = 8.7 Hz, ArH), 7.60 (1H, dd, *J* = 9.2, 3.0 Hz, ArH), 7.44 (1H, d, *J* = 15.3 Hz, C=CH), 7.25 (1H, m, ArH), 7.00 (3H, m, ArH), 3.87 (3H, s, –OCH_3_); ^13^C-NMR (CDCl_3_, 75 MHz) δ ppm 192.46 (d, *J* = 2.7 Hz, ArH), 161.98, 159.38 (d, *J* = 1.0 Hz, ArH), 156.12 (d, *J* = 238.7 Hz, ArH), 145.94, 129.48, 126.80, 123.44 (d, (d, *J* = 23.6 Hz, ArH), 119.51 (d, *J* = 7.3 Hz, ArH), 119.4 (d, *J* = 6.3 Hz, ArH), 116.67, 114.28, 113.99, 56.17.
(*E*)-3-(benzo[d][1,3]dioxol-5-yl)-1-(5-fluoro-2-hydroxyphenyl)prop-2-en-1-one (**4b**): yellow amorphous powder. Yield 76.51%. Mp 168–171 °C. R*_f_* = 0.72. ^1^H-NMR (CDCl_3_, 300 MHz) δ ppm 12.61 (1H, s, OH), 7.89 (1H, d, *J* = 15.3 Hz, C=CH), 7.58 (1H, dd, *J* = 9.2, 2.8 Hz, ArH), 7.38 (1H, d, *J* = 15.3 Hz, C=CH), 7.27 (1H, m, 1H), 7.21–7.15 (2H, m, ArH), 7.01 (1H, dd, *J* = 9.1, 4.6 Hz, ArH), 6.88 (1H, d, *J* = 7.9 Hz, ArH), 6.05 (2H, s, –OCH_2_O); ^13^C-NMR (CDCl_3_, 75 MHz) δ ppm 192.38 (d, *J* = 2.7 Hz, ArH), 159.40 (d, *J* = 1.1 Hz, ArH), 156.12 (d, J = 238.2 Hz), 150.28, 148.29, 145.94, 128.54, 125.75, 123.57 (d, J = 23.4 Hz), 119.56 (d, J = 7.3 Hz), 117.10, 114.29 (d, J = 23.4 Hz), 108.51, 106.51, 101.55.
(E)-3-(4-(dimethylamino)phenyl)-1-(5-fluoro-2-hydroxyphenyl)prop-2-en-1-one (**4c**): red crystals. Yield 95.69%. Mp 186–189 °C. R*_f_* = 0.48. ^1^H-NMR (CDCl_3_, 300 MHz) δ ppm 12.96 (1H, s, OH), 7.94 (1H, d, *J* = 15.1 Hz, C=CH), 7.56 (3H, d, *J* = 8.7 Hz, ArH), 7.31 (1H, d, *J* = 15.1 Hz, C=CH), 7.22–7.15 (1H, m, ArH), 6.98 (1H, dd, *J* = 4.5, 9.1 Hz, ArH), 6.69 (2H, d, *J* = 8.5 Hz, ArH), 3.04 (6H, s, –OCH_3_); ^13^C-NMR (CDCl_3_, 75 MHz) δ ppm 192.10 (d, *J* = 2.8 Hz), 159.26, 156.04 (d, *J* = 237.0 Hz), 152.18, 147.11, 130.77, 122.77 (d, *J* = 23.7 Hz), 121.70, 119.65 (d, *J* = 6.1 Hz), 119.27 (d*, J* = 7.7 Hz), 114.15 (d, *J* = 23.3 Hz), 113.15, 111.46, 39.74.
3-hydroxy-2-(4-methoxyphenyl)-4H-chromen-4-one (**f12a**): yellow amorphous powder. Yield 71.34%. Mp 233–235 °C. R*_f_* = 0.39. ^1^H-NMR (DMSO-*d_6_*, 500 MHz) δ ppm 9.24 (1H, s, OH), 8.20 (2H, dt, *J* = 8.9, 3.0, 2.1 Hz, ArH), 8.12 (1H, dd, *J =* 6.3, 1.5 Hz, ArH), 7.77 (1H, dt, *J =* 7.8, 1.0 Hz, ArH), 7.72 (1H, dd, J = 8.6, 1.0 Hz, ArH), 7.45 (1H, dt, *J =* 7.8, 1.0 Hz, ArH), 7.12 (2H, dt, *J* = 8.9, 3.0, 2.1 Hz, ArH), 3.85 (3H, s, –OCH_3_); ^13^C-NMR (DMSO-*d_6_*, 125 MHz)) δ ppm 172.48, 160.37, 154.35, 145.52, 137.95, 133.25, 129.24, 124.57, 124.26, 123.48, 121.23, 118.09, 113.91, 55.21.
2-(benzo[d][1,3]dioxol-5-yl)-3-hydroxy-4H-chromen-4-one (**f12b**): yellow amorphous powder. Yield 69.87%. Mp 213–215 °C. R*_f_* = 0.39. ^1^H-NMR (DMSO-*d_6_*, 300 MHz) δ ppm 9.52 (1H, s, OH), 8.10 (1H, d, *J* = 8.1 Hz, ArH), 7.84 (1H, dd, *J* = 8.5, 1.6 Hz, ArH), 7.78–7.73 (3H, m, ArH), 7.47 (1H, td, *J* = 8.1, 6.4, 1.7 Hz, ArH), 7.11 (1H, d, *J* = 8.3 Hz, ArH), 6.12 (2H, s, –OCH_2_O); ^13^C-NMR (DMSO-*d_6_*, 75 MHz) δ ppm 172.68, 154.40, 148.54, 147.52, 145.10, 138.33, 133.54, 125.05, 124.69, 122.76, 121.25, 118.36, 108.43, 107.52, 101.67.
2-(4-(dimethylamino)phenyl)-3-hydroxy-4H-chromen-4-one (**f12c**): Orange crystals. Yield 79.40%. Mp 182–183 °C. R*_f_* = 0.39. ^1^H-NMR (DMSO-*d_6_*, 500 MHz) δ ppm 9.16 (1H, s, OH), 8.12 (1H, d, *J* = 8.8 Hz, ArH), 8.09 (1H, dd, *J* = 7.8, 1.4 Hz, ArH), 7.69–7.75 (2H, m, ArH), 7.44 (1H, t, *J* = 7.3, 7.4 Hz, ArH), 6.83 (2H, d, *J* = 8.8 Hz, ArH), 2.99 (6H, s, –N(CH_3_)_2_); ^13^C-NMR (DMSO-*d_6_*, 125 MHz) δ ppm 171.98, 154.29, 151.03, 146.85, 137.29, 133.09, 128.99, 124.65, 124.32, 121.45, 118.15, 117.93, 111.40, 39.64.
6-chloro-3-hydroxy-2-(4-methoxyphenyl)-4H-chromen-4-one (**f13a**): yellow amorphous powder. Yield 86.42%. Mp 207–208 °C. R*_f_* = 0.52. ^1^H-NMR (DMSO-*d_6,_* 500 MHz) δ ppm 9.44 (1H, s, OH), 8.19 (2H, dt, *J* = 9.2, 2.9 Hz, ArH), 8.02 (1H, t, *J* = 1.4 Hz, ArH), 7.79 (2H, d, *J* = 1.8 Hz, ArH), 7.12 (2H, dt, *J* = 9.2, 2.9 Hz, ArH), 3.85 (3H, s, –OCH_3_); ^13^C-NMR (DMSO-*d_6_*, 125 MHz) δ ppm 171.42, 160.54, 152.86, 146.13, 138.12, 133.11, 129.37, 128.78, 123.18, 122.39, 120.64, 113.94, 55.25.
6-chloro-2-(4-(dimethylamino)phenyl)-3-hydroxy-4H-chromen-4-one (**f13c**): Orange crystals. Yield 78.67%. Mp 235–238 °C. R*_f_* = 0.57. ^1^H-NMR (DMSO-*d_6,_* 500 MHz) δ ppm 8.98 (1H, s, OH), 8.11 (2H, dt, *J* = 9.1, 3.0 Hz, ArH) 7.99 (1H, t, *J* = 1.7 Hz, ArH), 7.73–7.71 (2H, m, ArH), 6.83 (2H, dt, *J* = 9.1, 3.0 Hz, ArH), 7.11 (1H, d, *J* = 8.3 Hz, ArH), 3.01 (6H, s, –N(CH_3_)_2_); ^13^C-NMR (DMSO-*d_6_*, 125 MHz) δ ppm 170.51, 152.57, 151.01, 147.28, 137.03, 132.45, 128.75, 128.44, 123.14, 122.36, 120.20, 117.40, 111.12, 39.25.

### 4.4. Screening the Inhibitory Activity of Compounds

The screening assays for the inhibitory activity of the compounds in the rCPB2.8, rCPB3, and rH84Y enzymes were performed using 100 mM sodium acetate buffer containing 5 mM EDTA, 100 mM NaCl, 0.01% Triton X-100, and 20% glycerol, at pH 5.5. Enzyme aliquots were pre-incubated with 5 mM DTT for 5 min at 37 °C. After checking the initial rate of the reaction corresponding to the control, the enzymatic rate was measured at two concentrations of compounds, 1 µM and 5 µM. Enzyme activity was monitored by hydrolysis of the substrate Z-FR-MCA by measuring the fluorescence at λ_Ex_ = 360 nm and λ_Em_ = 480 nm on a Hitachi F2700 spectrofluorometer, obtaining the rate values in UAF/min (arbitrary fluorescence units by minute).

### 4.5. Determination of IC_50_ Values for Inhibitors

Cysteine proteases rCPB2.8, rCPB3, and rH84Y were assayed in 100 mM sodium acetate buffer containing 5 mM EDTA, 100 mM NaCl, 0.01% Triton X-100, and 20% glycerol, at pH 5.5. The enzymes were pre-incubated in the presence of 5 mM DTT for 5 min at 37 °C in a 1 mL final volume with constant stirring. Enzyme activities were monitored using fluorogenic probe Z-FR-AMC (9.25 µM final concentration), and the fluorescence was monitored by spectrofluorometry using fluorometer F2700 (Hitachi, Tokyo, Japan) set to λ_Ex_ = 360 nm and λ_Em_ = 480 nm. The IC_50_ evaluation was performed using a progressive increase in the concentration of the compounds; the IC_50_ values were calculated using nonlinear regression, and the data were analysed by Grafit 5.0.13 software using Equation (1).

(1) $$y=\frac{100\%}{1+{\left(\frac{x}{{\mathrm{IC}}_{50}}\right)}^{S}}.$$

### 4.6. Enzyme Kinetics and Determination of the Mechanism of Inhibition

**Figure 8:**
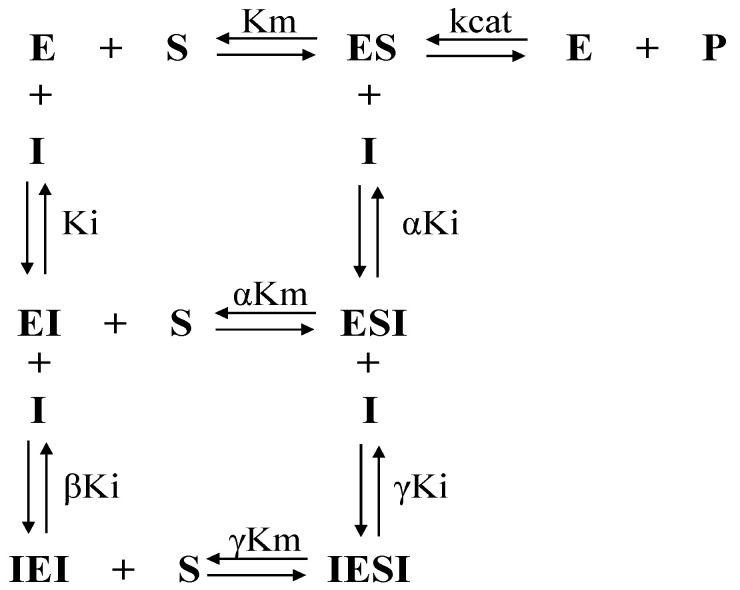
General mechanism of inhibition of cooperativity involving binding of a compound to the free enzyme E and the ES complex.

Studies of cysteine proteases rCPB2.8, rCPB3, and rH84Y inhibition kinetics were performed in different concentrations of Z-FR-AMC in the presence and absence of compounds using 100 mM sodium acetate buffer containing 5 mM EDTA, 100 mM NaCl, 0.01% Triton X-100, and 20% glycerol, at pH 5.5. Aliquots of the enzymes were pre-incubated with 5 mM DTT for 5 min at 37 °C. For every kinetic measurement, the compounds were pre-incubated with each enzyme for 10 min before adding the substrate. All kinetic assays were performed in duplicate. Inhibition constants were determined using different equations, depending on the inhibition mechanism. The assumed K_M_ values of rCPB2.8, rCPB3, and rH84Y for Z-FR-AMC were 3.23 µM, 2.99 µM, and 2.80 µM, respectively. The data of the activity rate and substrate concentration generated rectangular hyperbolic profiles that were linearised using the Lineweaver–Burk double-reciprocal plot. The replot profiles of the slope vs. inhibitor and intercept vs. inhibitor provided Ki and αKi parameters, respectively. If the replots present a parabolic profile, the system involves the participation of a second molecule of the compound, which can bind the enzyme complexed to the first compound (EI), forming IEI, and bind to the ESI complex, forming IESI. Linearisation is required, generating the 1/K_slope_ vs. inhibitor and 1/K_intercept_ vs. inhibitor replots, according to Equations (2) and (3).

(2) $$\frac{1}{K_{\mathrm{slope}}}=\frac{1}{K_{i}}+\frac{\left[I\right]}{{\beta K}_{i}^{2}},$$

(3) $$\frac{1}{K_{\mathrm{intercept}}}=\frac{1}{{\alpha K}_{i}}+\frac{\left[I\right]}{{\alpha \gamma K}_{i}^{2}},$$

where Ki is the inhibitory constant, [I] is the concentration of inhibitor, α is the factor of formation of the ESI complex (enzyme–substrate–inhibitor complex), β is the factor of formation of the IEI complex (inhibitor–enzyme–inhibitor complex), and γ is the factor of formation of the IESI complex (inhibitor–enzyme–substrate–inhibitor complex) according to the general mechanism (Figure 8). Furthermore, β and γ measure the cooperativity between the binding of the first and second inhibitor molecules to form IEI and IESI, respectively.

### 4.7. Molecular Modelling

Compounds **3c**, **f12a**, and **f12b** had their 3D structure drawn using the program MarvinSketch 16.9.5 (ChemAxon Ltd., Budapest, Hungary). The optimisation was carried out using the PM7 semiempirical method incorporated in the software MOPAC2016 [61]. A pH of 7.4 was considered for the definition of charges. 

The three-dimensional structure of rCPB2.8 was obtained through the homology modelling methodology using the Swiss-Model program [62]. Therefore, we used the 3D structure of papain-like cysteine protease obtained from the Protein Data Bank (PDB ID: 1F2A) as a layout [50] and the primary structure of rCPB2.8 as the target sequence. The choice of the crystal was based on the similarity with rCPB2.8, as well as that between the tested compound and the crystallographic ligand. To determine the potential binding modes at the active site of rCPB2.8, different binding poses were obtained on the basis of the overlay between the tested molecules and the crystallographic ligand. For the compounds with non-competitive inhibition with a positive cooperativity mechanism, we manually added a second molecule in different positions. The 3D structures of rCPB3 and rH84Y were obtained according to the amino-acid residues differences at their active sites, as described in the literature, using the program UCSF Chimera [63]. All the binding poses were further optimised geometrically.

Geometry optimisations were made using the GROMACS 2018 package [64] and the CHARMM force field [65]. The ligand topology was obtained from the Swiss Param Server [66], and the properties of the solvent were mimetic based on the TIP3P water model. A cubic box was used to guarantee a space of 1.2 nm between the protein and the box walls, and ions were under physiological conditions (0.15 µM) in order to neutralise the system charges. The energy optimisation steps were performed using the steepest descent followed by the conjugated gradient algorithm. The convergent criterion was a maximum force of 50 N on the atoms. The potential binding energy was measured, and the position of the binding mode with the best result was used to analyse the interactions with the binding site of all isoforms. 

### 4.8. Cytotoxicity on Mammalian Cells

The cytotoxic effect of the test samples was evaluated on NIH/3T3 fibroblasts, obtained from the Rio de Janeiro Cell Bank (Brazil). Cells were seeded in 96-well plates (5 × 10^5^ cells/well). After 24 h of fixation, cells were incubated for 48 h with test samples at 0.25–250 µg/mL, in triplicate. The tested compounds were dissolved in DMSO (Sigma-Aldrich^®^ SP/Brazil) while ensuring that the final concentration of the latter (0.25% at the highest sample concentration) did not interfere with cell viability. Doxorubicin (0.025–25 µg/mL was used as a positive control. Cells were fixed by adding 20% trichloroacetic acid and were subsequently stained with sulphorhodamine B (0.1%) diluted in acetic acid after 48 h of exposure [67]. Absorbance values were read on the PT-READER microplate instrument (Thermoplate^®^), and growth percentages were calculated according to procedures in the literature [68]. Cytotoxicity activity was expressed as the concentration of drug that inhibited cell growth by 50% (GL_50_), and growth was determined by nonlinear regression using Origin 6.0 software (OriginLab). The statistical significance was analysed using an unpaired Student’s *t*-test or a one-way analysis of variance. A *p*-value < 0.05 was considered statistically relevant. In addition, the SI was calculated using the ratio between cytotoxicity in NHI-3T3 cells (GL_50_) and activity in the parasite forms (IC_50_).

### 4.9. Parasites

A standard strain of *Leishmania (Leishmania amazonensis)* (IFLA/BR/1967/PH8) was used for the evaluation of in vitro antileishmanial activity. The promastigote forms were grown in Schneider’s insect medium (Sigma-Aldrich^®^, SP/Brazil) supplemented with 20% foetal bovine serum (Sigma-Aldrich^®^, SP/Brazil), 10,000 U/mL penicillin, and 10 mg/mL streptomycin (Sigma-Aldrich^®^, SP/Brazil). Skin lesions were routinely isolated from previously induced skin lesions in BALB/c mice and kept in axenic culture until the 20th serial passage.

### 4.10. Antipromastigote Assay

Promastigote forms of *L. amazonensis* in the log growth phase (10^e6^ parasites/mL) were added to 96-well plates and incubated in sextuplicate with the test samples (0.25–10.0 µg/mL). The microplates were incubated at 26 °C for 72 h. Cell viability was assessed by adding 5 mg/mL of MTT ([3-(4,5-dimethylthiazol-2-yl)-2,5-diphenyltetrazolium bromide] (Sigma-Aldrich^®^ SP/Brazil) [20]. Results were expressed as the half-maximal inhibitory concentration (IC_50_) calculated by a nonlinear dose–response regression curve using the statistical program GraphPad PRISM 5.0. Pentamidine (Sigma-Aldrich ^®^ SP/Brazil; 0.25–10 µg/mL) was used as a positive control, and pure Schneider culture medium was used as a negative control.

## Figures and Tables

**Table 1:** Reaction yields of chalcone and flavonol-like compounds.

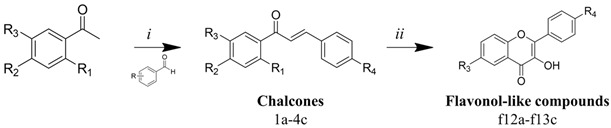					
Entry	R_1_	R_2_	R_3_	R_4_	Yield (%)
1	H	Cl	H	–OCH_3_	67.11 (**1a**)
2	H	Cl	H	–OCH_2_O	86.19 (**1b**)
3	H	Cl	H	–N(CH_3_)_2_	73.26 (**1c**)
4	OH	H	H	–OCH_3_	76.26 (**2a**)
5	OH	H	H	–OCH_2_O	71.43 (**2b**)
6	OH	H	H	–N(CH_3_)_2_	82.34 (**2c**)
7	OH	H	Cl	–OCH_3_	90.40 (**3a**)
8	OH	H	Cl	–OCH_2_O	86.70 (**3b**)
9	OH	H	Cl	–N(CH_3_)_2_	81.74 (**3c**)
10	OH	H	F	–OCH_3_	84.32 (**4a**)
11	OH	H	F	–OCH_2_O	76.51 (**4b**)
12	OH	H	F	–N(CH_3_)_2_	95.69 (**4c**)
13	-	H	H	–OCH_3_	71.34 (**f12a**)
14	-	H	H	–OCH_2_O	69.87 (**f12b**)
15	-	H	H	–N(CH_3_)_2_	79.40 (**f12c**)
16	-	H	Cl	–OCH_3_	86.42 (**f13a**)
17	-	H	Cl	–N(CH_3_)_2_	78.67 (**f13c**)

*i:* NaOH (3 M), EtOH (dry); *ii*: H_2_O_2_/NaOH (3 M), EtOH (dry).

**Table 2:** Chalcone inhibitory potential (IC_50_) of chalcones to modulate the enzyme activity of *Leishmania mexicana* cysteine proteases rCPB2.8, rCPB3, and rH84Y.

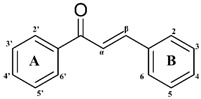				
Entry	Compound	IC_50_ (µM)		
		rCPB2.8	rCPB3	rH84Y
1	**1a**	13.66 ± 0.74	9.91 ± 0.59	29.74 ± 2.62
2	**1b**	7.42 ± 0.25	5.60 ± 0.35	15.34 ± 0.60
3	**1c**	2.75 ± 0.18	10.17 ± 0.52	11.70 ± 1.27
4	**2a**	2.35 ± 0.19	7.14 ± 0.86	16.34 ± 0.46
5	**2b**	20.63 ± 1.06	10.99 ± 0.40	4.67 ± 0.13
6	**2c**	11.74 ± 0.38	8.96 ± 0.61	9.90 ± 0.48
7	**3a**	7.11 ± 0.32	4.22 ± 0.81	6.84 ± 0.26
8	**3b**	18.37 ± 1.22	11.56 ± 0.77	8.23 ± 0.21
9	**3c**	3.97 ± 0.08	5.47 ± 0.59	4.81 ± 0.32
10	**4a**	3.35 ± 0.17	4.51 ± 0.19	20.34 ± 1.78
11	**4b**	6.61 ± 0.41	17.27 ± 1.46	7.77 ± 0.72
12	**4c**	9.88 ± 0.73	37.97 ± 7.24	4.34 ± 0.43

**Table 3:** Flavonol-like inhibition potential (IC_50_) in modulating the enzyme activity of *Leishmania mexicana* cysteine proteases rCPB2.8, rCPB3, and rH84Y.

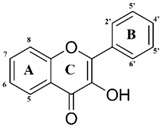				
Entry	Compound	IC_50_ (µM)		
		rCPB2.8	rCPB3	rH84Y
13	**f12a**	4.72 ± 0.38	7.71 ± 0.78	3.85 ± 0.27
14	**f12b**	5.23 ± 0.32	7.06 ± 0.63	8.85 ± 0.33
15	**f12c**	10.32 ± 0.39	16.67 ± 2.53	28.56 ± 1.62
16	**f13a**	1.88 ± 0.07	10.56 ± 1.11	11.71 ± 1.54
17	**f13c**	14.18 ± 0.65	29.75 ± 2.08	20.35 ± 1.02

**Table 4:** Chalcone and flavonoid affinity constants and inhibition mechanisms of enzymes rCPB2.8, rCPB3, and rH84Y.

rCPB2.8								
Compound	Ki (µM)	αKi (µM)	βKi (µM)	γKi (µM)	α	β	γ	Mechanism
**3c**	100 ± 18	147 ± 28	0.55 ± 0.10	1.15 ± 0.21	1.47	0.0055	0.0115	NCPC
**f12a**	12.4 ± 1.4	10.7 ± 1.1	3.65 ± 0.39	0.25 ± 0.03	1	0.29	0.02	NCPC
**f12b**	39.2 ± 4.1	24.9 ± 2.3	1.17 ± 0.12	0.51 ± 0.09	0.634	0.029	0.013	NCPC
**rCPB3**								
**3c**	15.4 ± 3.0	141 ± 27	2.21 ± 0.16	0.77 ± 0.14	9.15	0.14	0.05	NCPC
**f12a**	7.47 ± 0.64	7.50 ± 0.66	---	---	1			SLNC
**f12b**	13.7 ± 1.4	13.7 ± 1.2	---	---	1			SLNC
**rH84Y**								
**3c**	29.4 ± 4.7	127 ± 20	1.45 ± 0.27	1.06 ± 0.18	4.31	0.049	0.036	NCPC
**f12a**	11.2 ± 0.8	11.2 ± 0.5	---	---	1			SLNC
**f12b**	6.08 ± 0.37	6.06 ± 0.24	---	---	1			SLNC

NCPC: non-competitive inhibition with positive cooperativity; SLNC: simple linear non-competitive inhibition.

**Table 5:** Calculated energy values for the interaction of **3c, f12a**, **and f12b** with the tested rCPB isoforms ^a^.

Enzyme	Binding-Free Energy	Δ_E_	Ki (µM)
**3c**			
rCPB2.8	−2.46	22.24	100 ± 18
rCPB3	−24.70	0	12.4 ± 1.4
rH84Y	−22.49	2.21	39.2 ± 4.1
**f12a**			
rCPB2.8	−3.58	2.07	12.4 ± 1.4
rCPB3	−5.65	0	7.47 ± 0.64
rH84Y	−5.24	0.42	11.2 ± 0.8
**f12b**			
rCPB2.8	−0.21	5.44	39.2 ± 4.1
rCPB3	−4.30	1.35	13.7 ± 1.4
rH84Y	−5.65	0	6.08 ± 0.37

^a^*:* All energy values are expressed in kcal·mol^−1^.

## Data Availability

All datasets generated for this study are included in the article/Supplementary Materials.

## Supplementary Materials

The following supporting information can be downloaded at https://www.mdpi.com/article/10.3390/microorganisms11010225/s1: Figures S1–S10; Tables S1–S3; Spectra 1–34. Figure S1: Determination of the affinity constants of the 3c compound in the inhibition of rCPB2.8; Figure S2: Determination of the affinity constants of the f12a compound in the inhibition of rCPB2.8; Figure S3: Determination of the affinity constants of the f12b compound in the inhibition of rCPB2.8; Figure S4: Determination of the affinity constants of the 3c compound in the inhibition of rCPB3; Figure S5: Determination of the affinity constants of the f12a compound in the inhibition of rCPB3; Figure S6: Determination of the affinity constants of the f12b compound in the inhibition of rCPB3; Figure S7: Determination of the affinity constants of the 3c compound in the inhibition of rH84Y; Figure S8: Determination of the affinity constants of the f12a compound in the inhibition of rH84Y; Figure S9: Determination of the affinity constants of the f12b compound in the inhibition of rH84Y; Table S1: Differences of amino acid residues at the active site of rCPB2.8, rCPB3 and rH84Y; Table S2: Potential energy values for the biding positions of compound 3c at the active site of rCPB2.8; Table S3: Potential energy values for the biding positions of compound f12a at the active site of rCPB3a; Figure S10: Cytotoxicity of all flavonoid derivatives against NHI-3T3 cells. The supplementary material comprises the results of the enzyme kinetics and determination of the mechanism of inhibition in vitro tests. Furthermore, we present the NMR spectra section of all synthetic compounds.
