# Supplementary material for: Flavonoid Derivatives as New Potent Inhibitors of Cysteine Proteases: An Important Step toward the Design of New Compounds for the Treatment of Leishmaniasis
Source: Microorganisms. 2023 Jan 16;11(1):225. doi: 10.3390/microorganisms11010225 (PMC9866541; doi:10.3390/microorganisms11010225)
Supplement: Supplementary file 1 [file microorganisms-11-00225-s001.zip › microorganisms-2069025-supplementary.pdf]

Supplementary Material

Flavonoid derivatives as new potent inhibitors of cysteine proteases: An important step toward the design of new compounds for the treatment of leishmaniasis

Estela Mariana Guimarães Lourenço[1], Juliana Fortes Di Iório[2], Fernanda da Silva[3], Felipe Leonardo Bley Fialho[1], Melquisedeque Mateus Monteiro[4], Adilson Beatriz[1], Renata Trentin Perdomo[4], Euzébio Guimarães Barbosa[5], Jean Pierre Oses,[6] Carla Cardozo Pinto de Arruda[3], Wagner Alves de Souza Júdice[2,*], Jamal Rafique[1,7*], Dênis Pires de Lima[1,*]

1 Laboratory of Synthesis and Transformation of Organic Molecules - SINTMOL, Institute of Chemistry, Universidade Federal de Mato Grosso do Sul, Av. Senador Filinto Muller, 1555, Campo Grande, MS, Brazil
2 Centro Interdisciplinar de Investigação Bioquímica (CIIB), Universidade de Mogi das Cruzes (UMC), Mogi das Cruzes, SP, Brazil
3 Laboratório de Parasitologia Humana, Instituto de Biociências, Universidade Federal de Mato Grosso do Sul, Campo Grande, MS, Brazil
4 Laboratory of Molecular Biology and Cell Culture, School of Pharmaceutical Sciences, Food Technology, and Nutrition, Universidade Federal de Mato Grosso do Sul, Campo Grande, MS, Brazil
5 Laboratório de Química Farmacêutica Computacional, Departamento de Farmácia, Universidade Federal do Rio Grande do Norte, Natal, Brazil
6 Laboratório de Neurociências, Instituto de Biociências, Universidade Federal do Rio Grande, Rio Grande, RS, Brazil.
7 Instituto de Química, Universidade Federal de Goiás - UFG, Goiânia, 74690-900, GO-Brazil

* Correspondence:
wagnerjudice@gmail.com (WASJ)
jamal.chm@gmail.com; jamal.rafique@ufms.br (JR)
denis.lima@ufms.br (DPL)

Summary

Figure S1: Determination of the affinity constants of the **3c** compound in the inhibition of rCPB2.8 3
Figure S2: Determination of the affinity constants of the **f12a** compound in the inhibition of rCPB2.8. 3
Figure S3: Determination of the affinity constants of the **f12b** compound in the inhibition of rCPB2.8. 4
Figure S4: Determination of the affinity constants of the **3c** compound in the inhibition of rCPB3. 4
Figure S5: Determination of the affinity constants of the **f12a** compound in the inhibition of rCPB3. 5
Figure S6: Determination of the affinity constants of the **f12b** compound in the inhibition of rCPB3. 5
Figure S7: Determination of the affinity constants of the **3c** compound in the inhibition of rH84Y. 6
Figure S8: Determination of the affinity constants of the **f12a** compound in the inhibition of rH84Y. 6
Figure S9: Determination of the affinity constants of the **f12b** compound in the inhibition of rH84Y. 7
Table S1: Differences of amino acid residues at the active site of rCPB2.8, rCPB3 and rH84Y. .... **Error! Bookmark not defined.**
Table S2: Potential energy values for the biding positions of compound **3c** at the active site of rCPB2.8 9
Table S3: Potential energy values for the biding positions of compound f12a at the active site of rCPB3a........ 9
Figure S10: Cytotoxicity of all flavonoid derivatives against NHI-3T3 cells........ 9
NMR spectra section 10

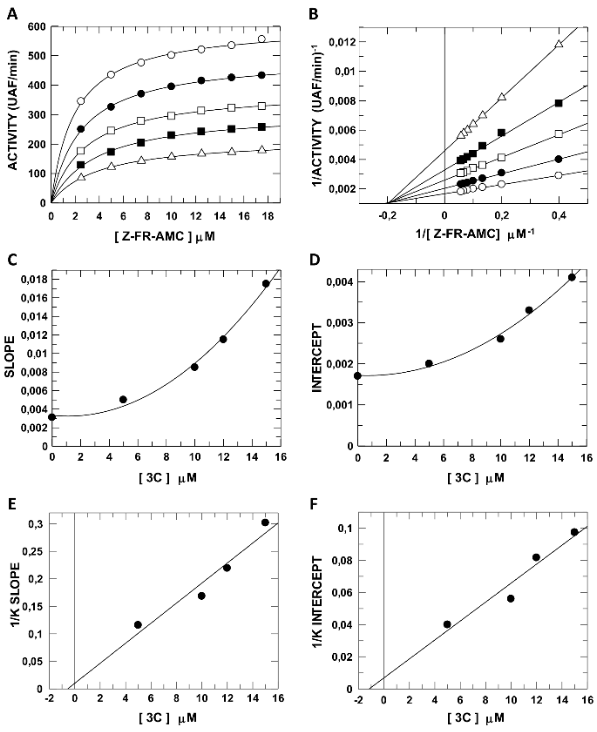

A: Michaelis-Menten plot; B: Lineweaver-Burk plot; C: Slope replot; D: Intercept replot; E: 1/K_{Slope} replot; F: 1/$K_{Intercept}$ replot. ○→ control; ●→ 5 µM; ●→ 10 µM; ■→ 12 µM; △→ 15 µM

Figure S1: Determination of the affinity constants of the **3c** compound in the inhibition of rCPB2.8

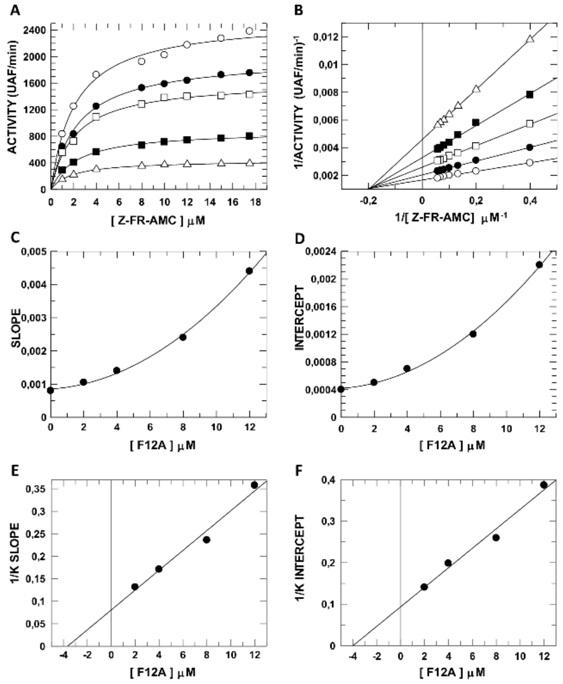

A: Michaelis-Menten plot; B: Lineweaver-Burk plot; C: Slope replot; D: Intercept replot; E: 1/K_{Slope} replot; F: 1/$K_{Intercept}$ replot. ○→ control; ●→ 2 µM; ●→ 4 µM; ■→ 8 µM; △→ 12 µM

Figure S2: Determination of the affinity constants of **f12a** compound in the inhibition of rCPB2.8.

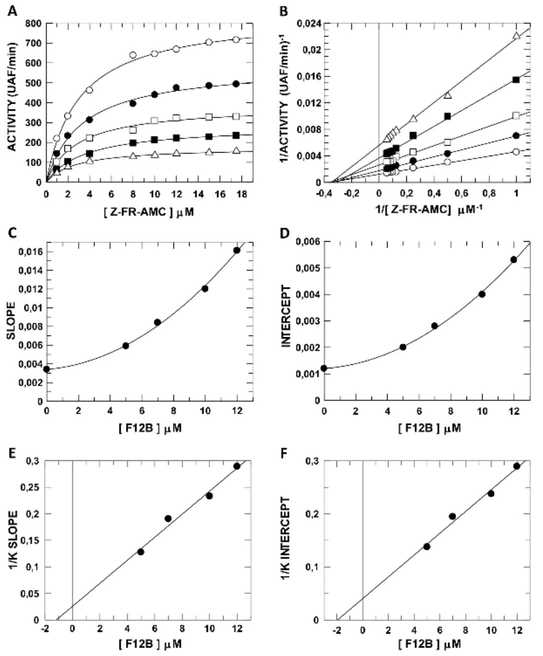

A: Michaelis-Menten plot; B: Lineweaver-Burk plot; C: Slope replot; D: Intercept replot; E: 1/K_{Slope} replot; F: 1/$K_{Intercept}$ replot. ○→ control; ●→ 5 μM; ●→ 7 μM; ■→ 10 μM; △→ 12 μM

Figure S3: Determination of the affinity constants of the **f12b** compound in the inhibition of rCPB2.8.

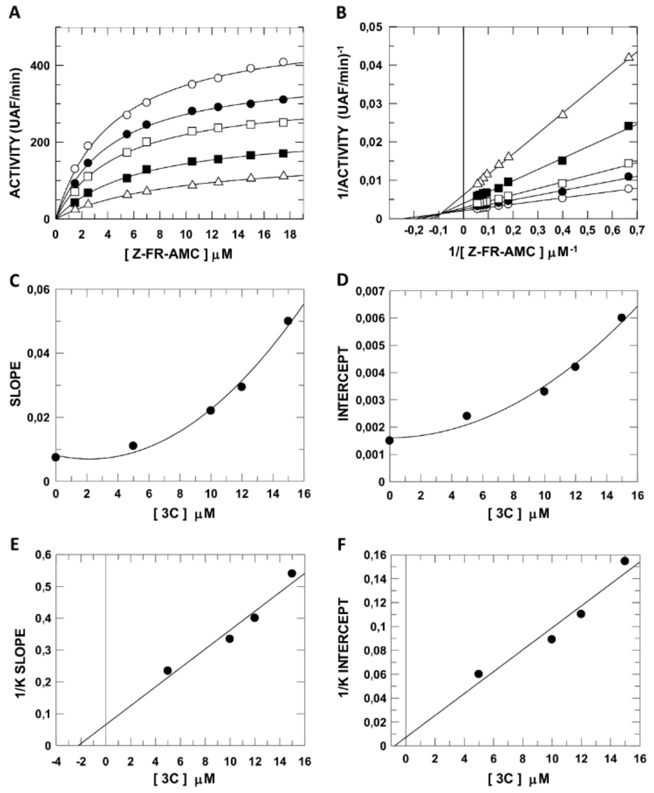

A: Michaelis-Menten plot; B: Lineweaver-Burk plot; C: Slope replot; D: Intercept replot; E: 1/K_{Slope} replot; F: 1/$K_{Intercept}$ replot. ○→ control; ●→ 5 μM; ●→ 10 μM; ■→ 12 μM; △→ 15 μM

Figure S4: Determination of the affinity constants of the **3c** compound in the inhibition of rCPB3.

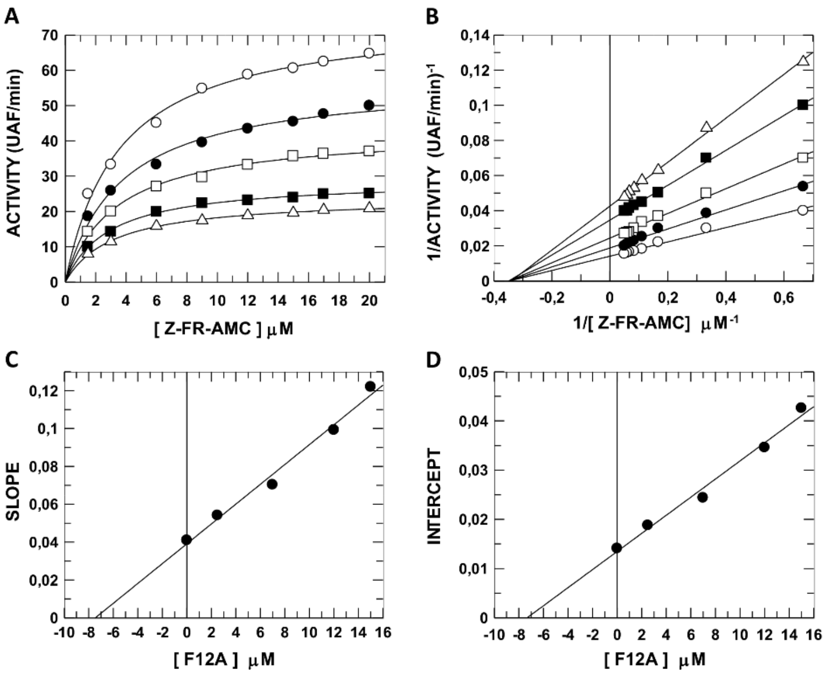

A: Michaelis-Menten plot; B: Lineweaver-Burk plot; C: Slope replot; D: Intercept replot; E: $1/K_{Slope}$ replot; F: $1/K_{Intercept}$ replot. ○→ control; ●→ 2.5 µM; ●→ 7 µM; ■→ 12 µM; △→ 15 µM

Figure S5: Determination of the affinity constants of the **f12a** compound in the inhibition of rCPB3.

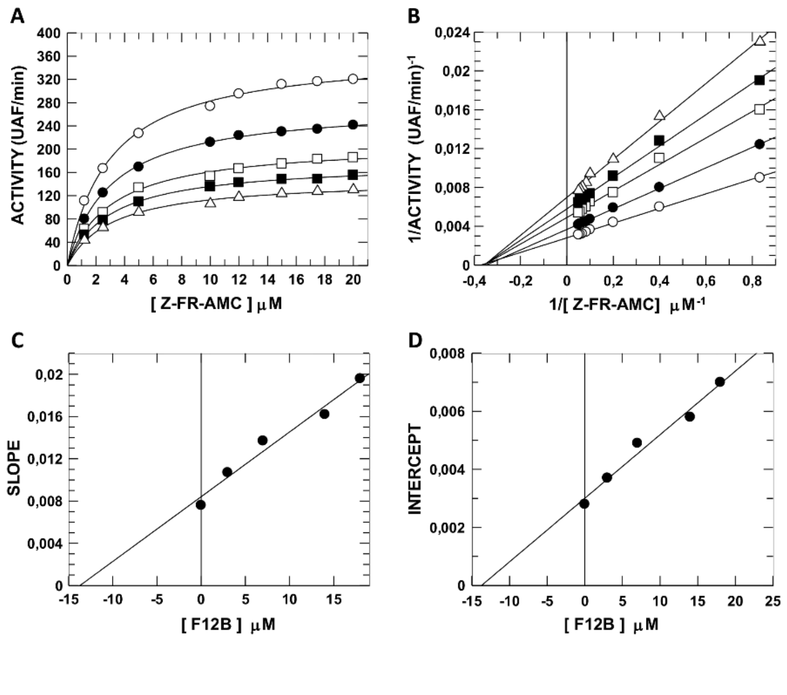

A: Michaelis-Menten plot; B: Lineweaver-Burk plot; C: Slope replot; D: Intercept replot; E: $1/K_{Slope}$ replot; F: $1/K_{Intercept}$ replot. ○→ control; ●→ 3 µM; ●→ 7 µM; ■→ 14 µM; △→ 18 µM

Figure S6: Determination of the affinity constants of the **f12b** compound in the inhibition of rCPB3.

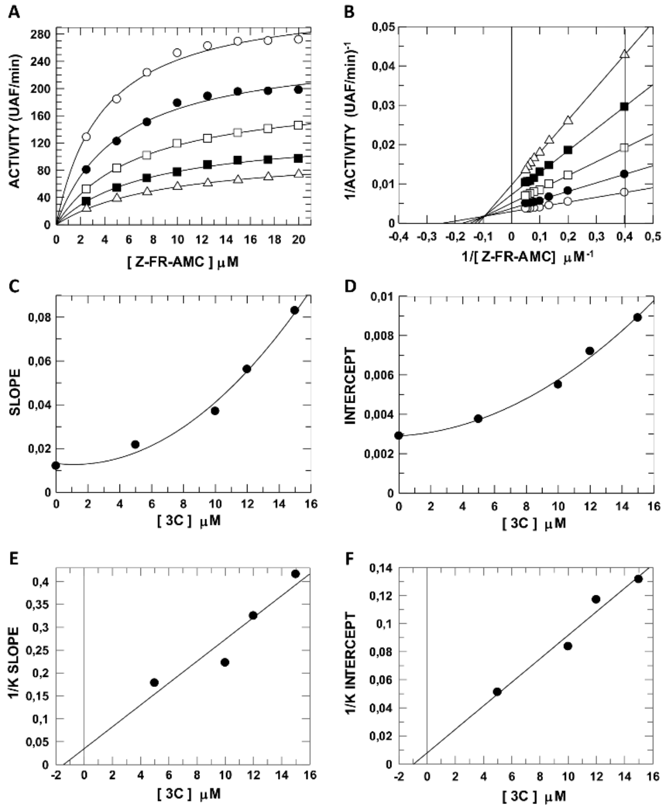

A: Michaelis-Menten plot; B: Lineweaver-Burk plot; C: Slope replot; D: Intercept replot; E: $1/K_{Slope}$ replot; F: $1/K_{Intercept}$ replot. ○→ control; ●→ 5 µM; ●→ 10 µM; ■→ 12 µM; △→ 15 µM

Figure S7: Determination of the affinity constants of the **3c** compound in the inhibition of rH84Y.

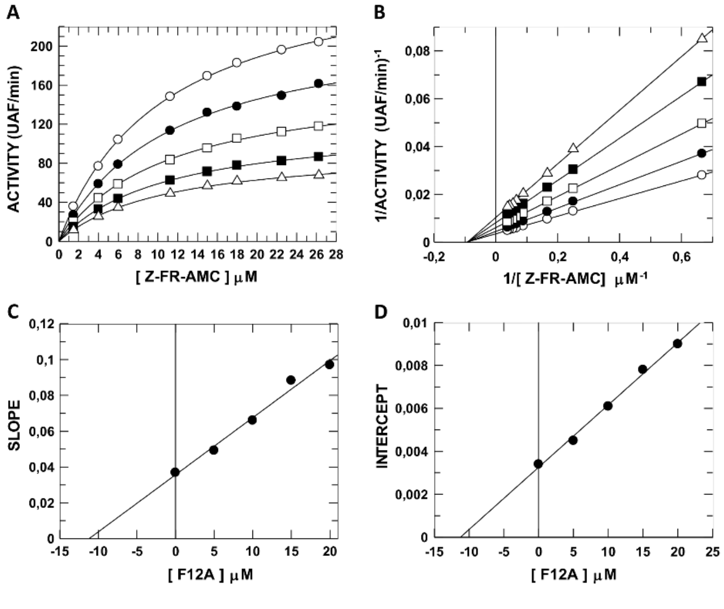

A: Michaelis-Menten plot; B: Lineweaver-Burk plot; C: Slope replot; D: Intercept replot; E: $1/K_{Slope}$ replot; F: $1/K_{Intercept}$ replot. ○→ control; ●→ 5 µM; ●→ 10 µM; ■→ 15 µM; △→ 20 µM

Figure S8: Determination of the affinity constants of the **f12a** compound in the inhibition of rH84Y.

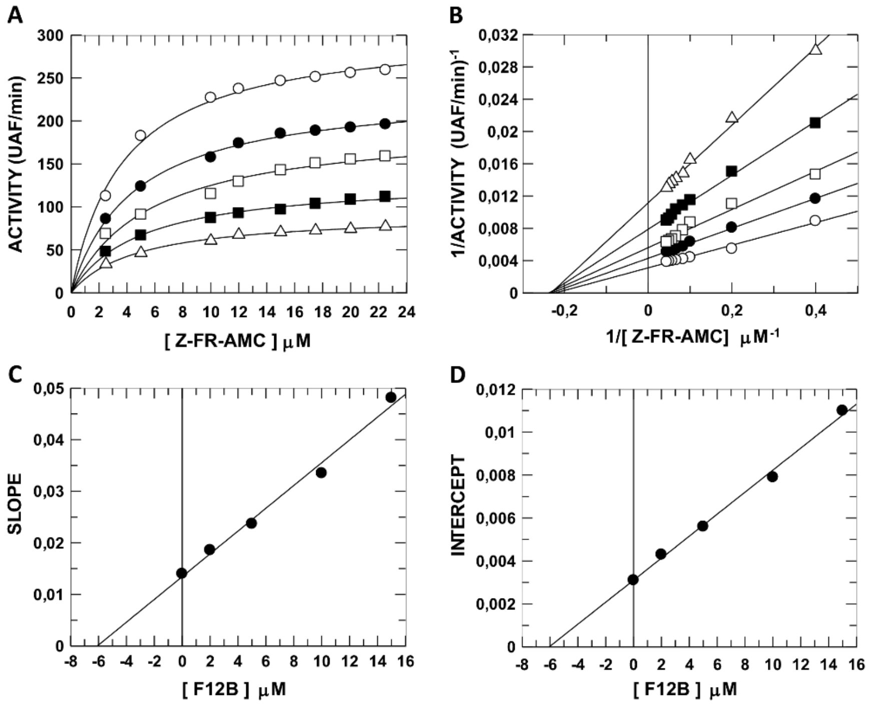

A: Michaelis-Menten plot; B: Lineweaver-Burk plot; C: Slope replot; D: Intercept replot; E: $1/K_{Slope}$ replot; F: $1/K_{Intercept}$ replot. ○→ control; ●→ 2 μM; ●→ 5 μM; ■→ 10 μM; △→ 15 μM

Figure S9: Determination of the affinity constants of the **f12b** compound in the inhibition of rH84Y.

Table S1: Differences of amino acid residues at the active site of rCPB2.8, rCPB3 and rH84Y

**Enzyme**[1]	**Amino acid residues of active site**[2]				
	**143**	**185**	**186**	**189**	**209**
rCPB2.8	ASN	ASP	ASP	ASP	HIS
rCPB3	ASP	ASN	SER	SER	HIS
rH84Y	ASP	ASN	SER	SER	TYR

[1]The number of amino acid residue may change depends on the crystal structure. [2]All of three isoforms are recombinant enzymes (r) lacking the C-terminal region.

Table S2: Potential energy values for the biding positions of compound **3c** at the active site of rCPB2.8[a]

Positions	Potential energy	Δ_{Energy}
1	-58282	4751
2	-105794	0
3	-90651	1514
4	-101704	409
5	-98316	747
6	-52679	5312
7	-50644	5515
8	-53940	51854

a: All values are expressed in kcal mol^{-1}

Table S3: Potential energy values for the biding positions of compound **f12a** at the active site of rCPB3[a]

Positions	Potential energy	Δ_{Energy}
1	-106212	586
2	-62477	44321
3	-106398	400
4	-97614	9184
5	-106798	0
6	-103814	2984
7	-103849	2949
8	-106176	622

a: All values are expressed in kcal mol^{-1}

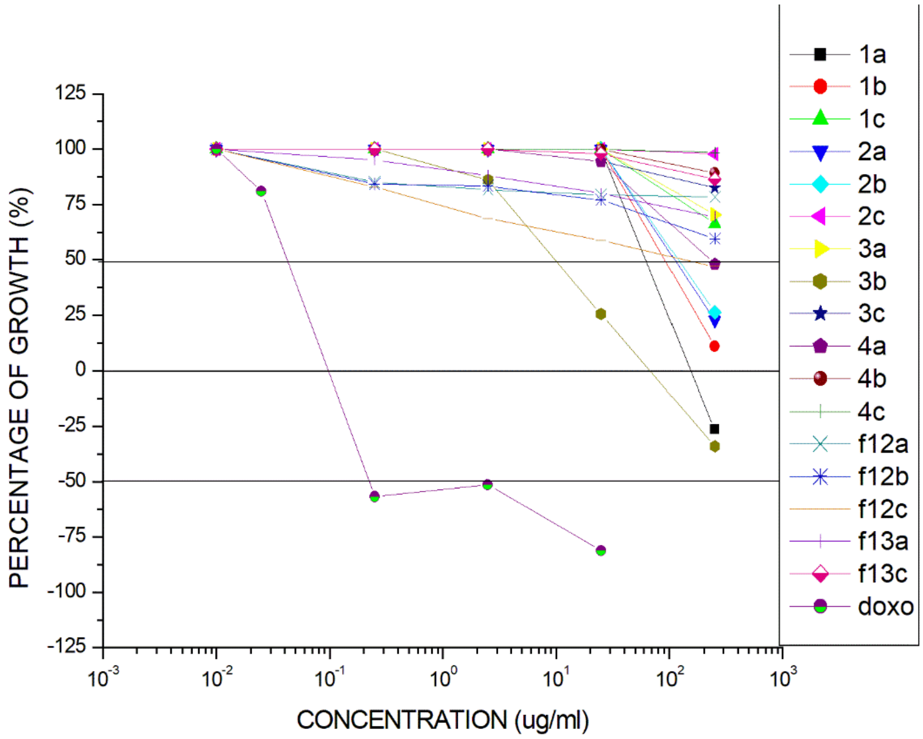

Figure S10: Cytotoxicity of all flavonoid derivatives against NHI-3T3 cells

NMR spectra section

Spectra 1: ^{1}H NMR of compound **1a** 11
Spectra 2: ^{13}C NMR of compound **1a** 11
Spectra 3: ^{1}H NMR of compound **1b** 12
Spectra 4: ^{13}C NMR of compound **1b** 12
Spectra 5: ^{1}H NMR of compound **1c** 13
Spectra 6: ^{13}C NMR of compound **1c** 13
Spectra 7: ^{1}H NMR of compound **2a** 14
Spectra 8: ^{13}C NMR of compound **2a** 14
Spectra 9: ^{1}H NMR of compound **2b** 15
Spectra 10: ^{13}C NMR of compound **2b** 15
Spectra 11: ^{1}H NMR of compound **2c** 16
Spectra 12: ^{13}C NMR of compound **2c** 16
Spectra 13: ^{1}H NMR of compound **3a** 17
Spectra 14: ^{13}C NMR of compound **3a** 17
Spectra 15: ^{1}H NMR of compound **3b** 18
Spectra 16: ^{13}C NMR of compound **3b** 18
Spectra 17: ^{1}H NMR of compound **3c** 19
Spectra 18: ^{13}C NMR of compound **3c** 19
Spectra 19: ^{1}H NMR of compound **4a** 20
Spectra 20: ^{13}C NMR of compound **4a** 20
Spectra 21: ^{1}H NMR of compound **4b** 21
Spectra 22: ^{13}C NMR of compound **4b** 21
Spectra 23: ^{1}H NMR of compound **4c** 22
Spectra 24: ^{13}C NMR of compound **4c** 22
Spectra 25: ^{1}H NMR of compound **f12a** 23
Spectra 26: ^{13}C NMR of compound **f12a** 23
Spectra 27: ^{1}H NMR of compound **f1b** 24
Spectra 28: ^{13}C NMR of compound **f12b** 24
Spectra 29: ^{1}H NMR of compound **f1c** 25
Spectra 30: ^{13}C NMR of compound **f12c** 25
Spectra 31: ^{1}H NMR of compound **f3a** 26
Spectra 32: ^{13}C NMR of compound **f13a** 26
Spectra 33: ^{1}H NMR of compound **f3c** 27
Spectra 34: ^{13}C NMR of compound **f13c** 27

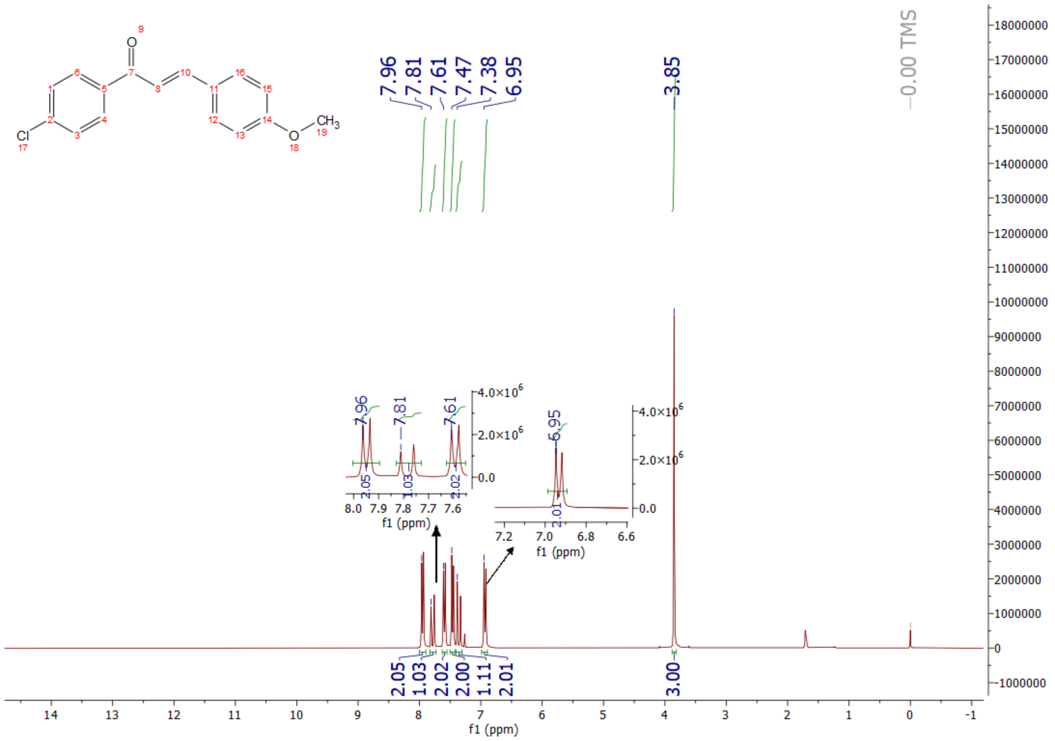

Spectra 1: ^{1}H NMR of compound **1a**

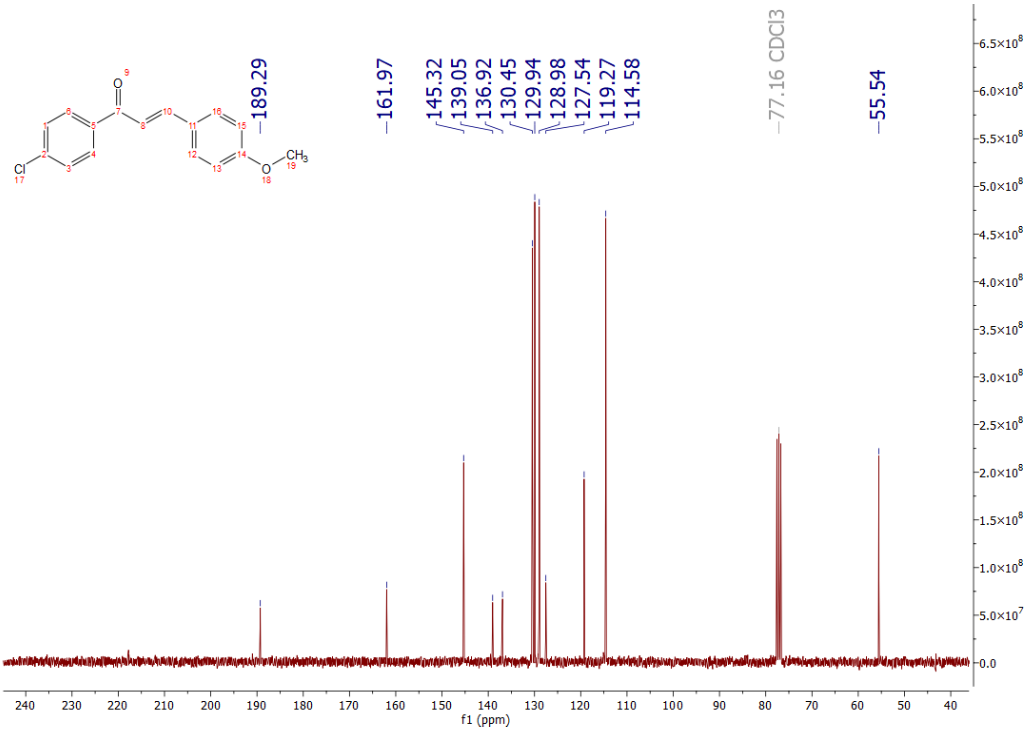

Spectra 2: ^{13}C NMR of compound **1a**

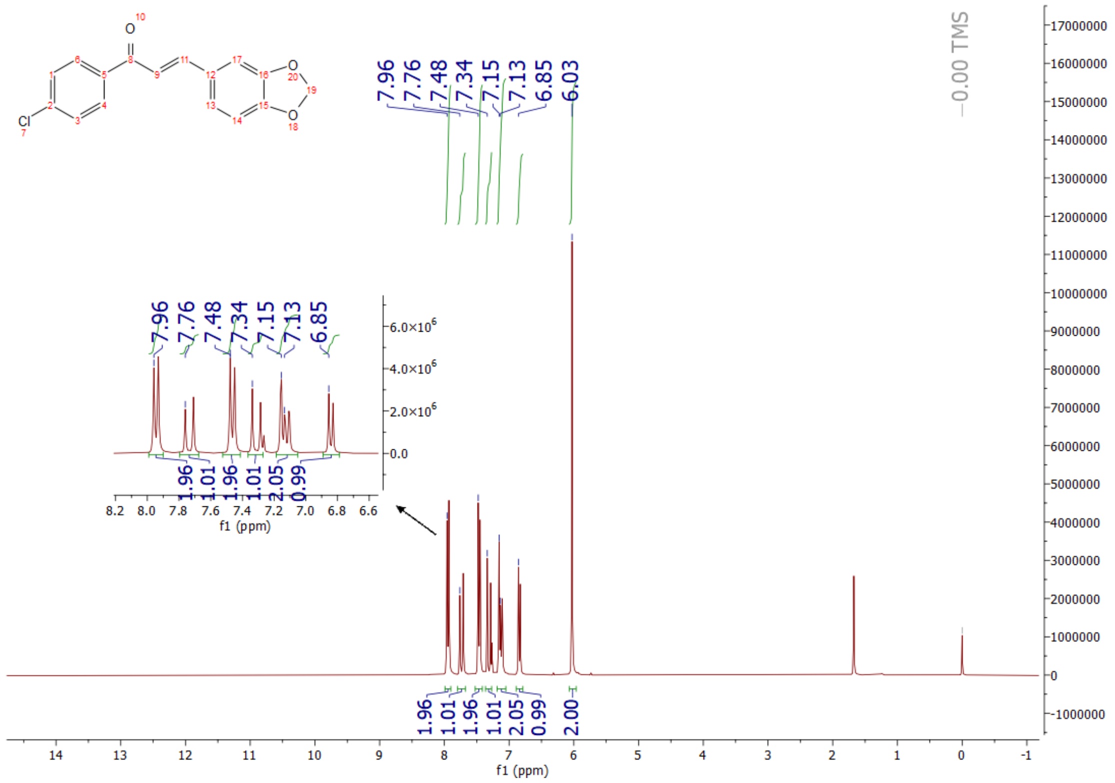

Spectra 3: ^{1}H NMR of compound **1b**

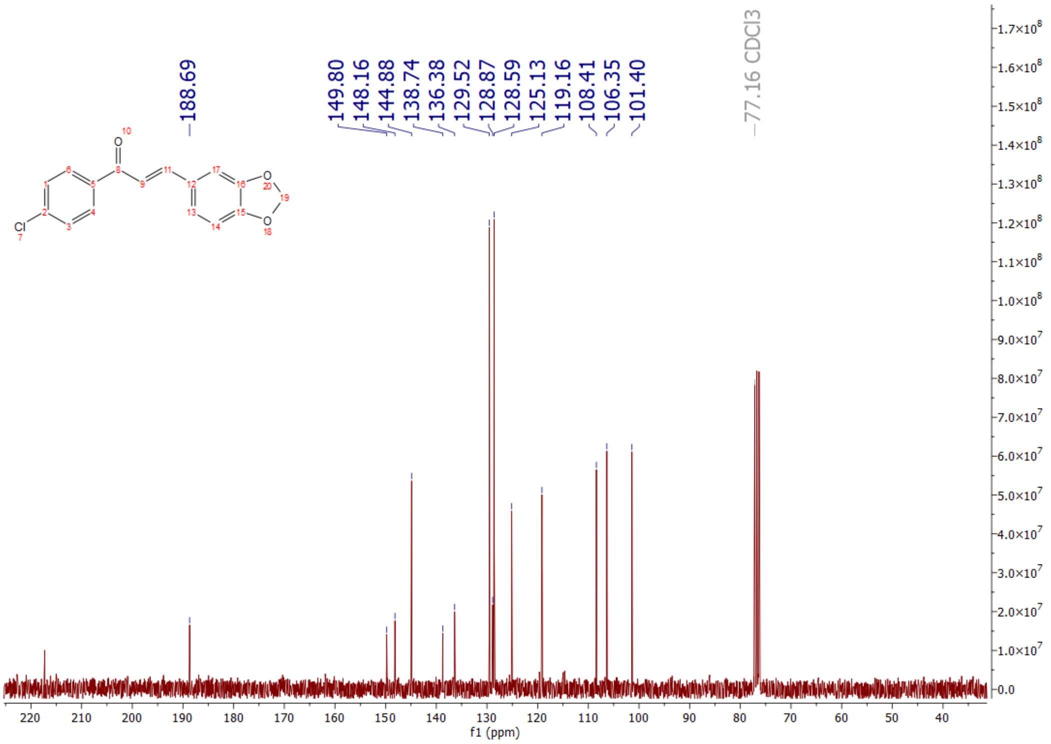

Spectra 4: ^{13}C NMR of compound **1b**

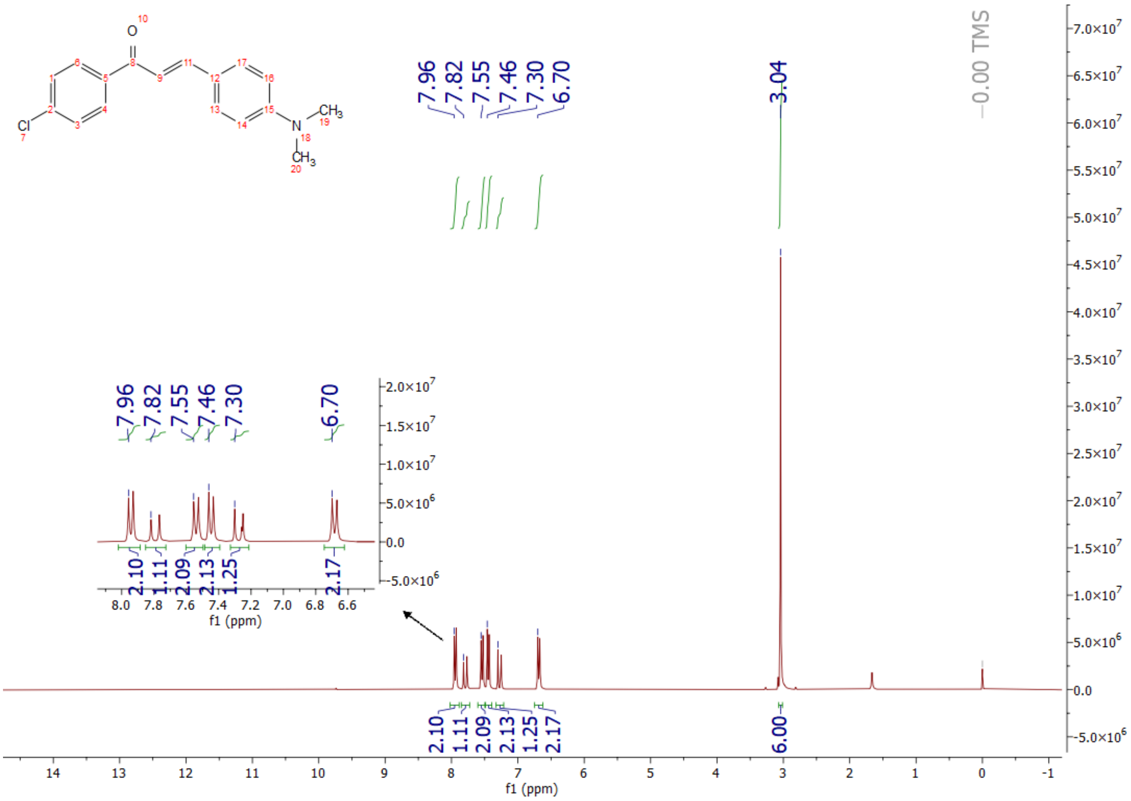

Spectra 5: ^{1}H NMR of compound **1c**

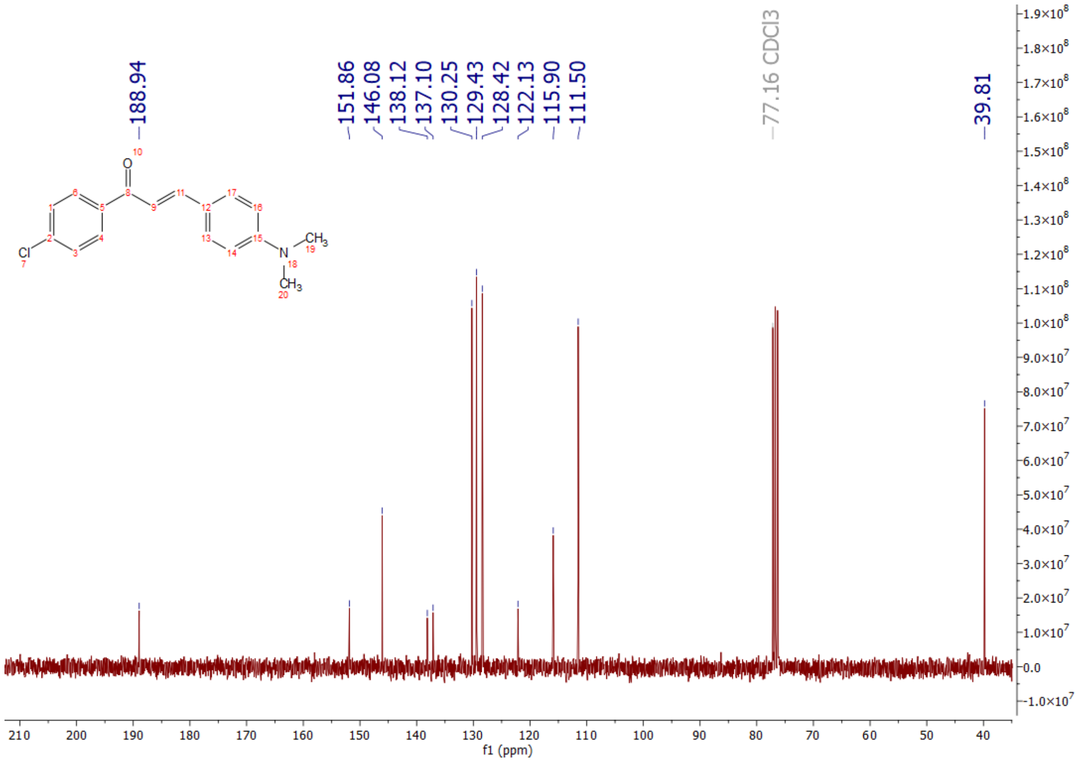

Spectra 6: ^{13}C NMR of compound **1c**

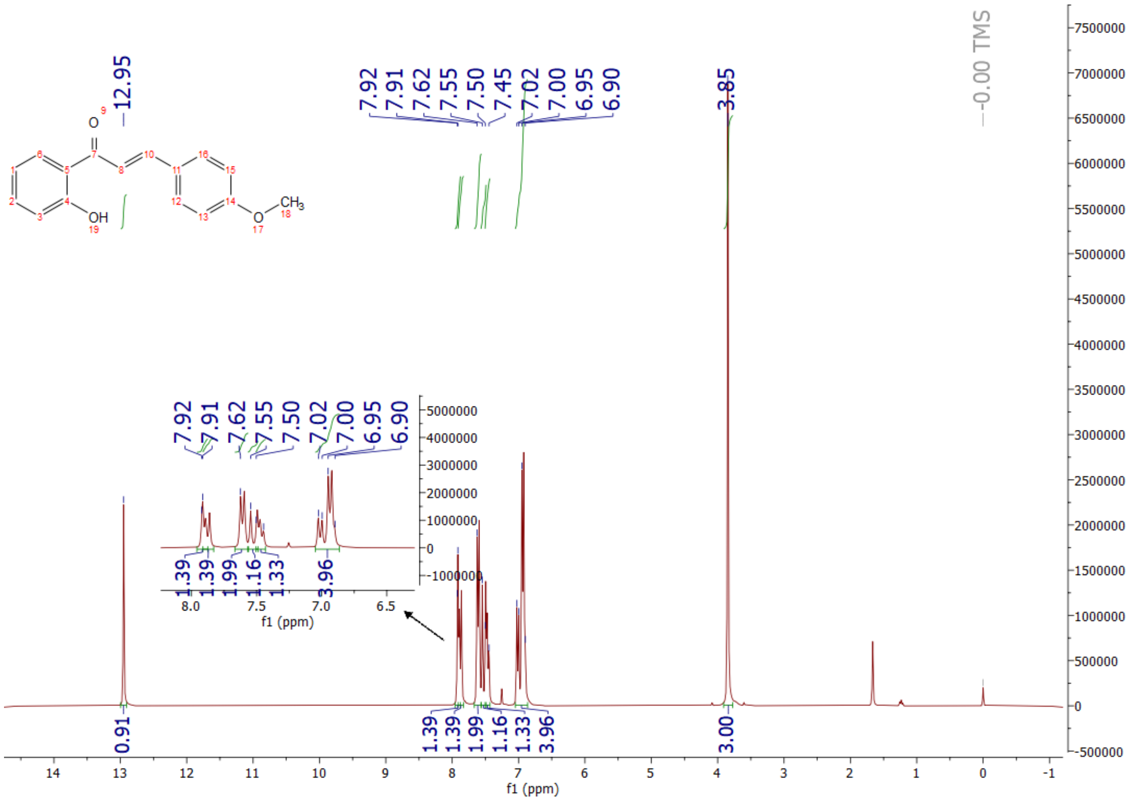

Spectra 7: ^{1}H NMR of compound **2a**

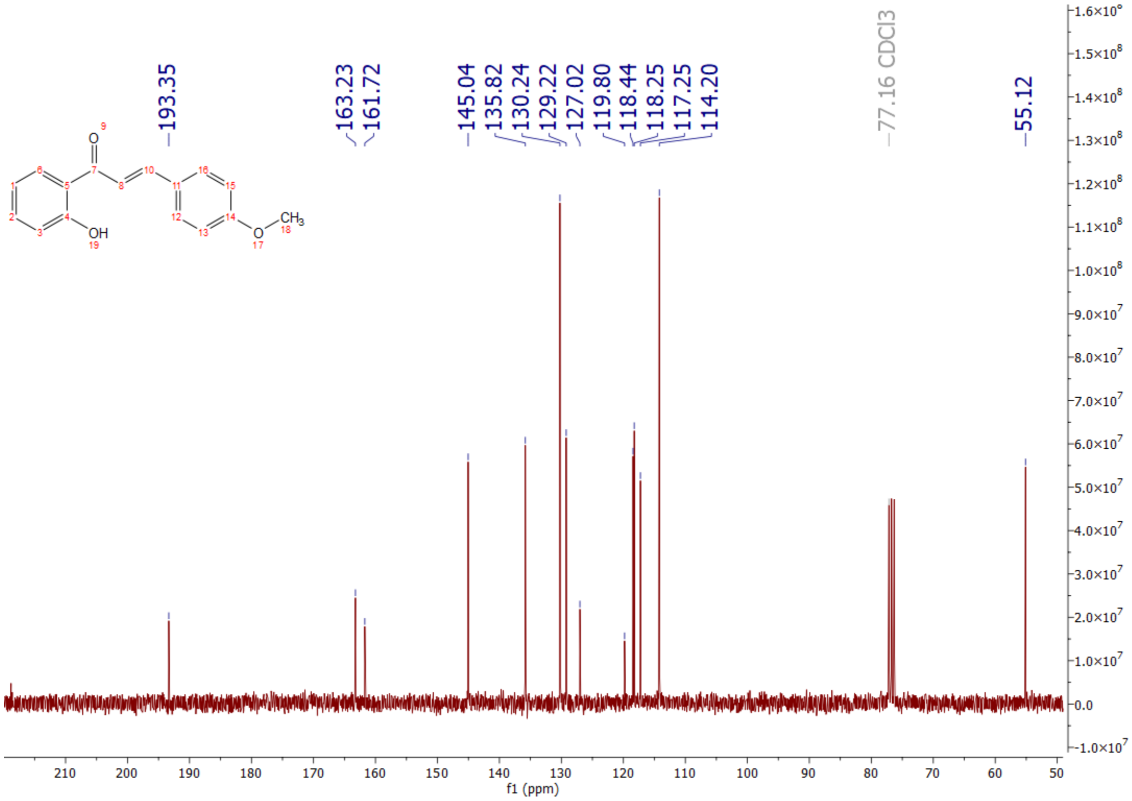

Spectra 8: ^{13}C NMR of compound **2a**

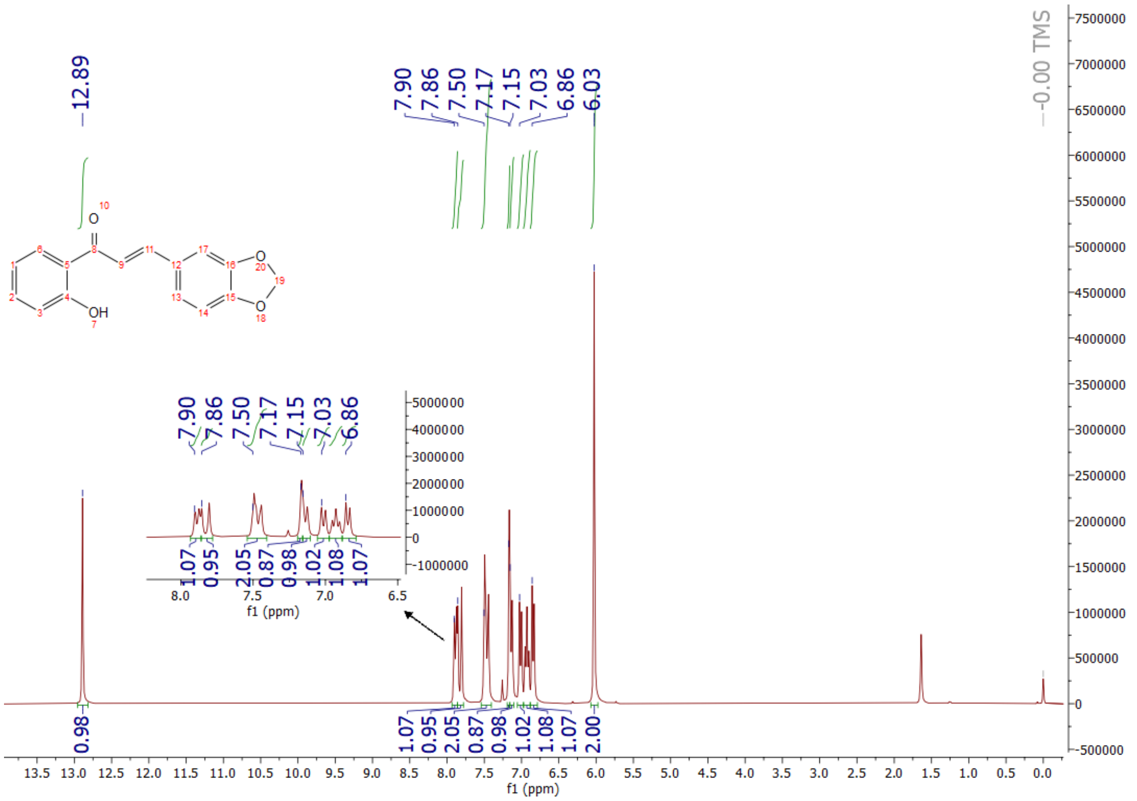

Spectra 9: ^{1}H NMR of compound **2b**

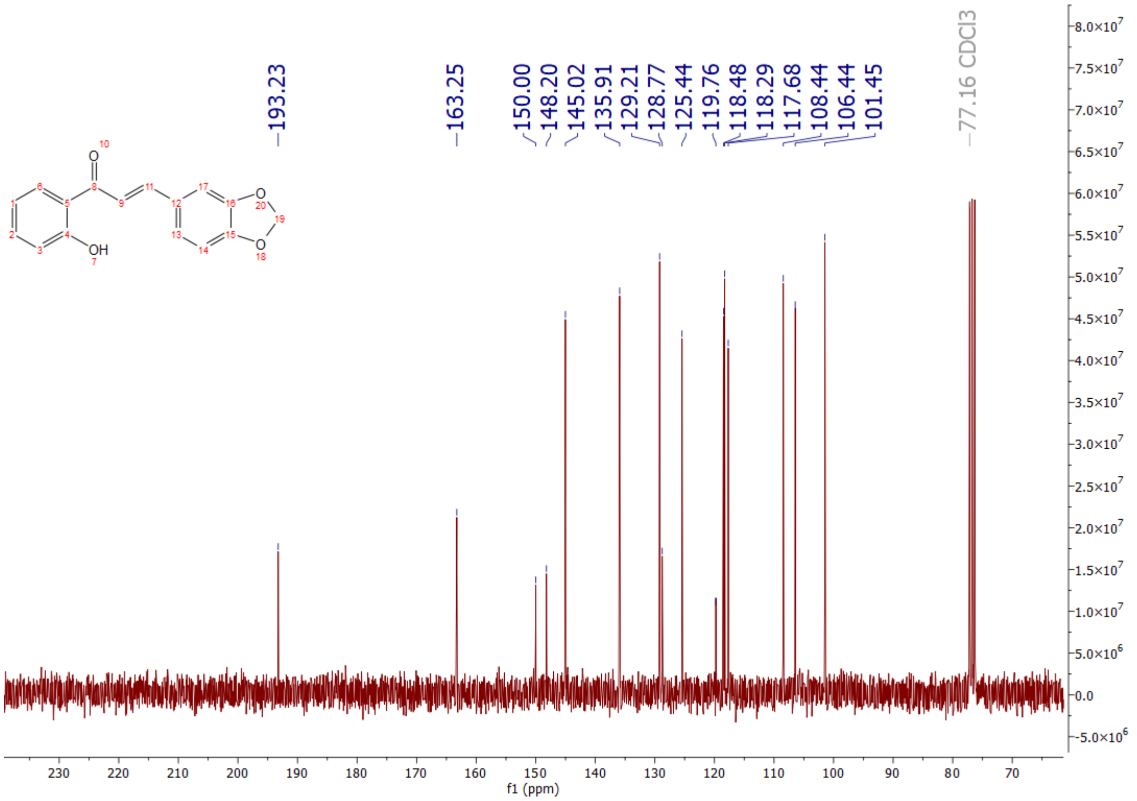

Spectra 10: ^{13}C NMR of compound **2b**

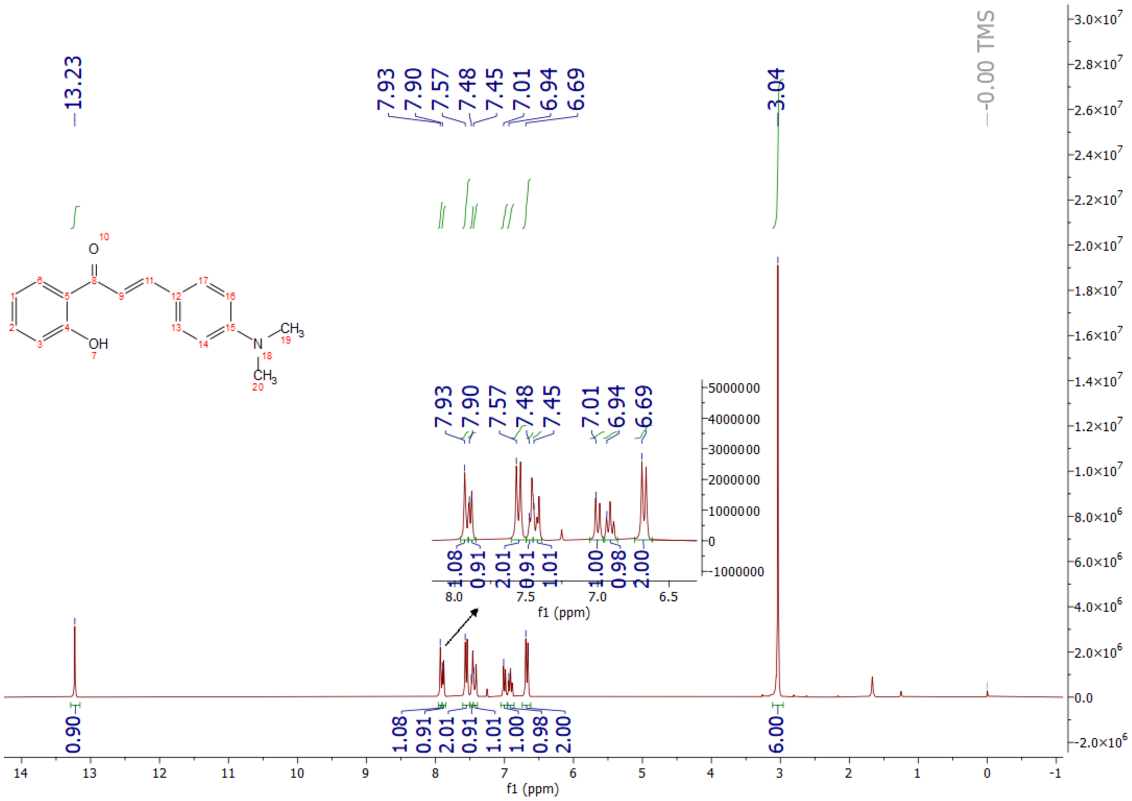

Spectra 11: ^{1}H NMR of compound **2c**

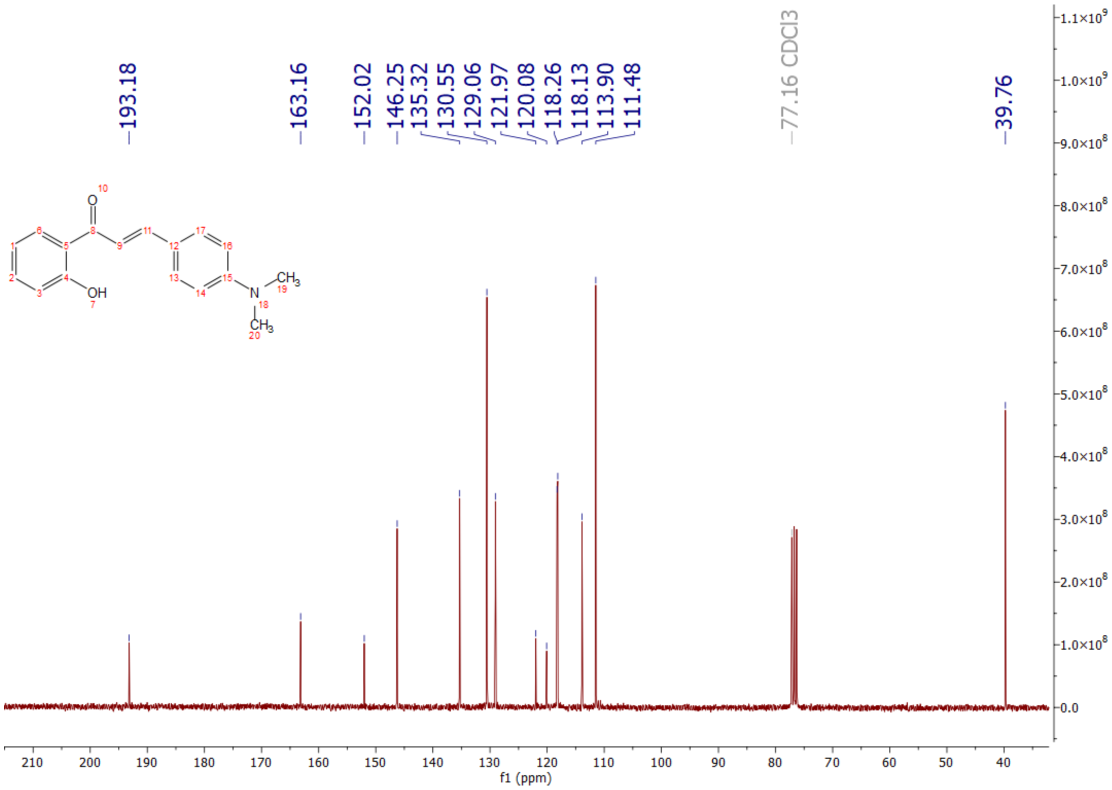

Spectra 12: ^{13}C NMR of compound **2c**

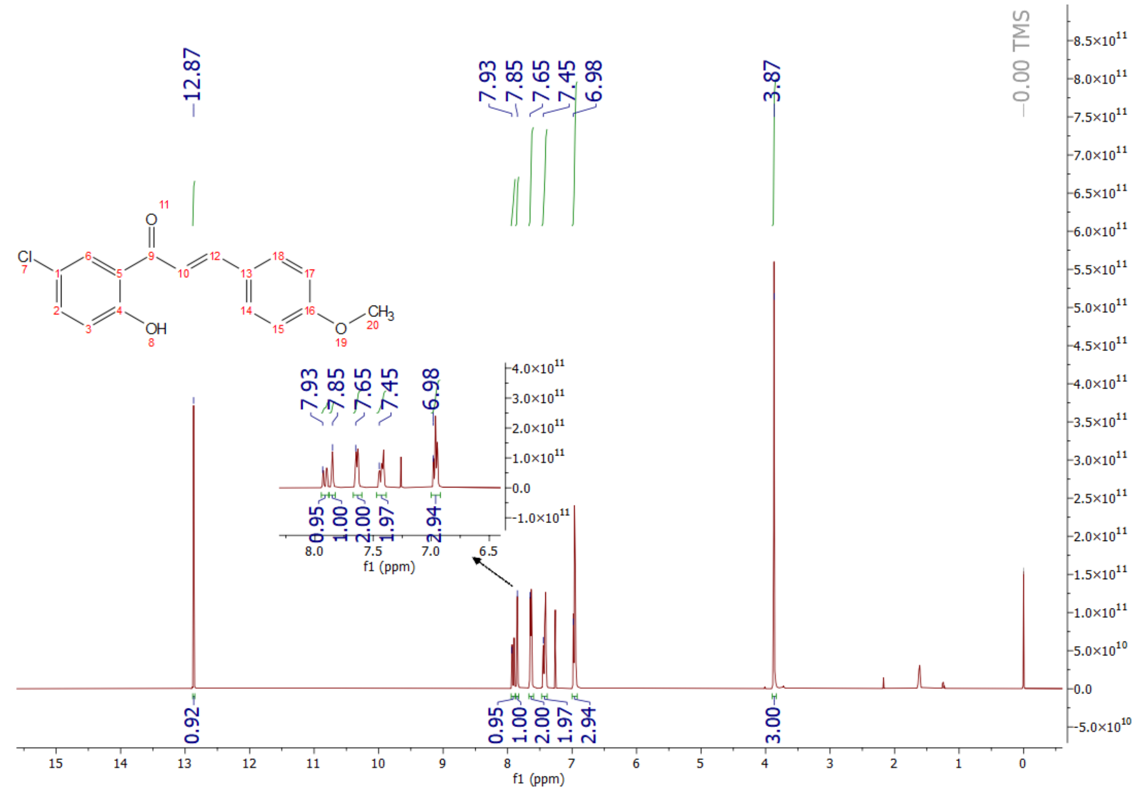

Spectra 13: ^{1}H NMR of compound **3a**

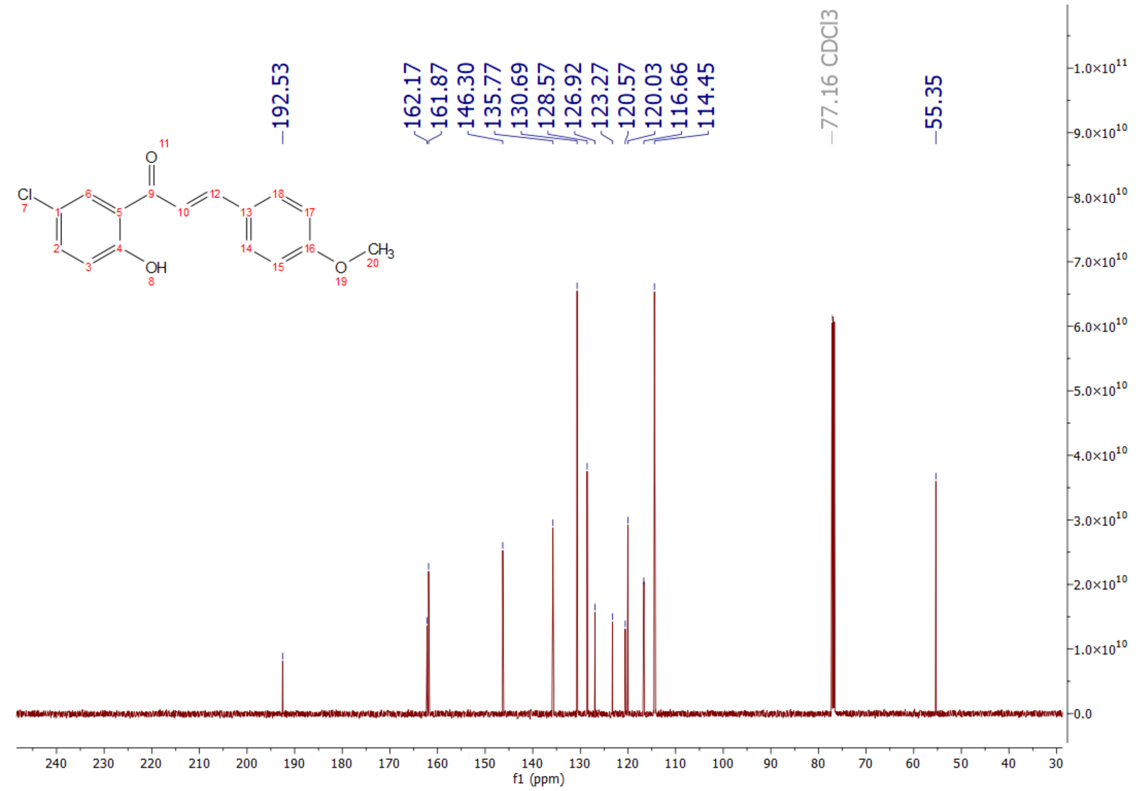

Spectra 14: ^{13}C NMR of compound **3a**

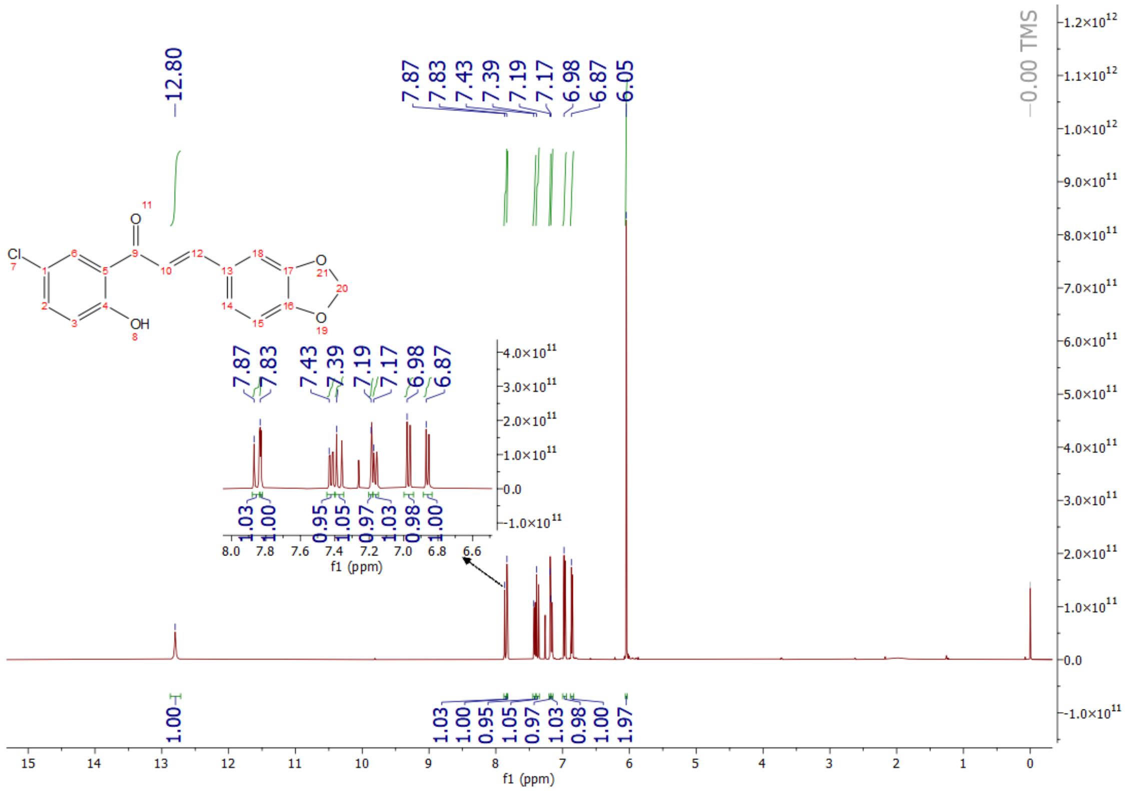

Spectra 15: ^{1}H NMR of compound **3b**

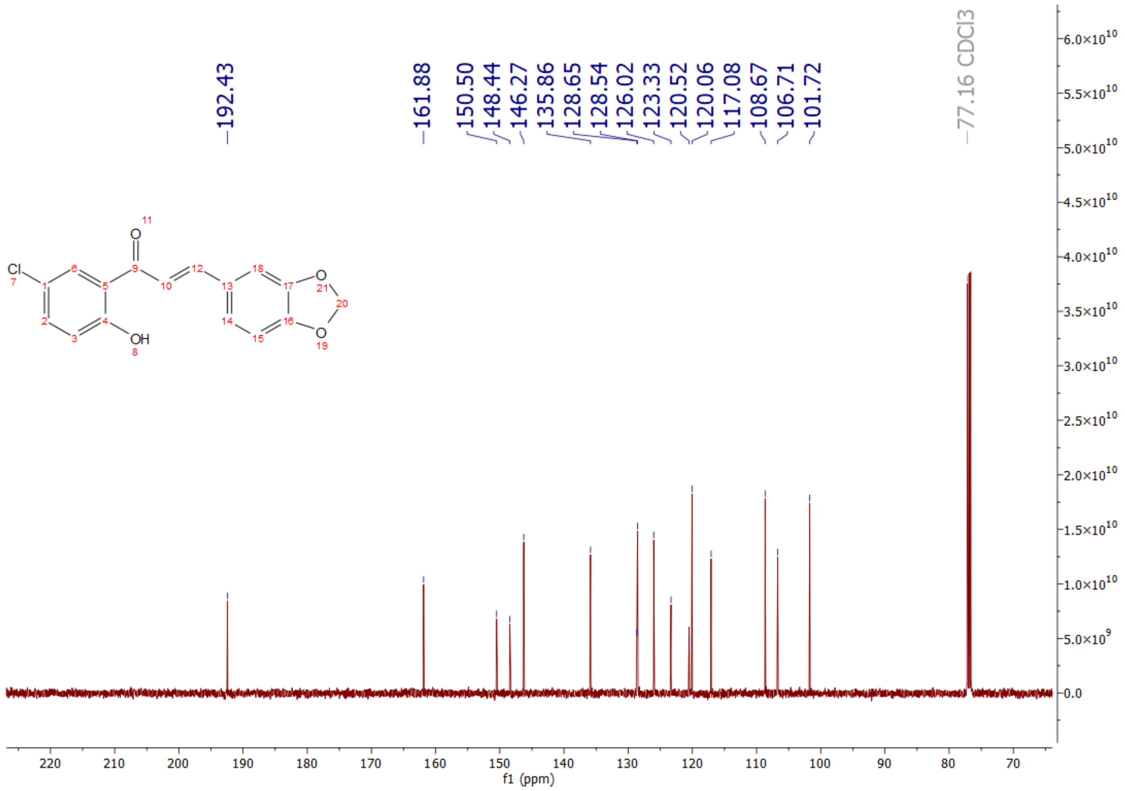

Spectra 16: ^{13}C NMR of compound **3b**

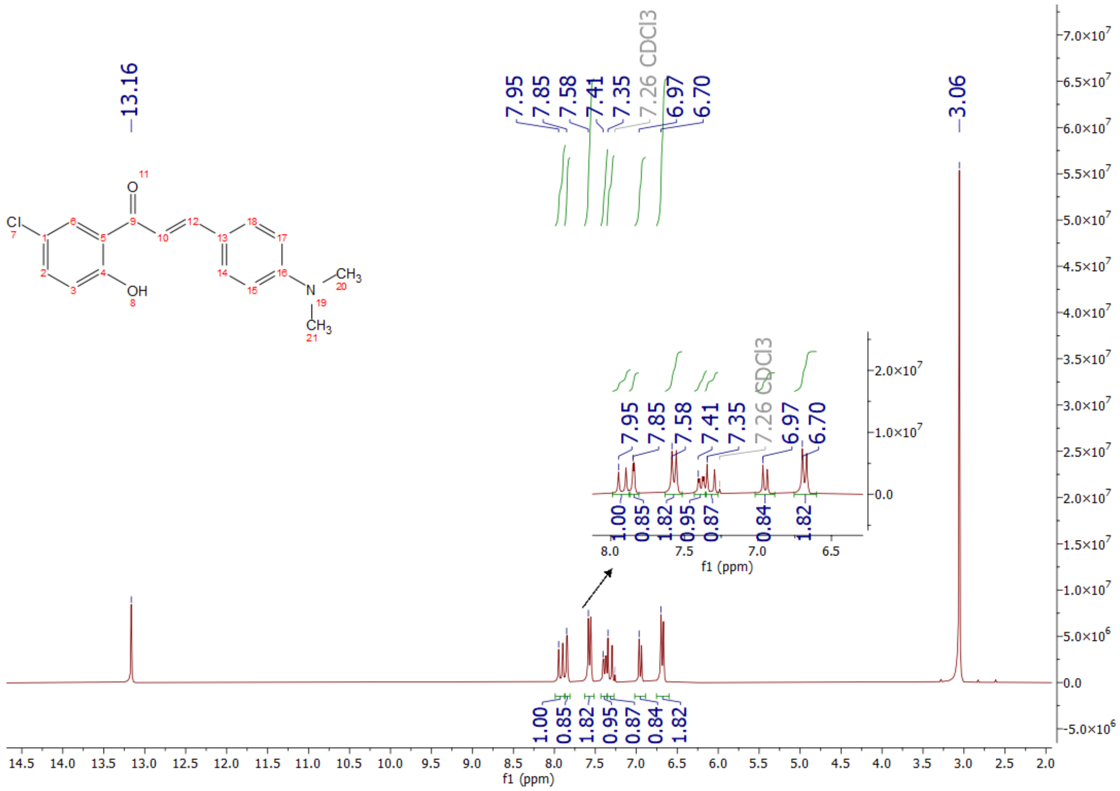

Spectra 17: ^{1}H NMR of compound **3c**

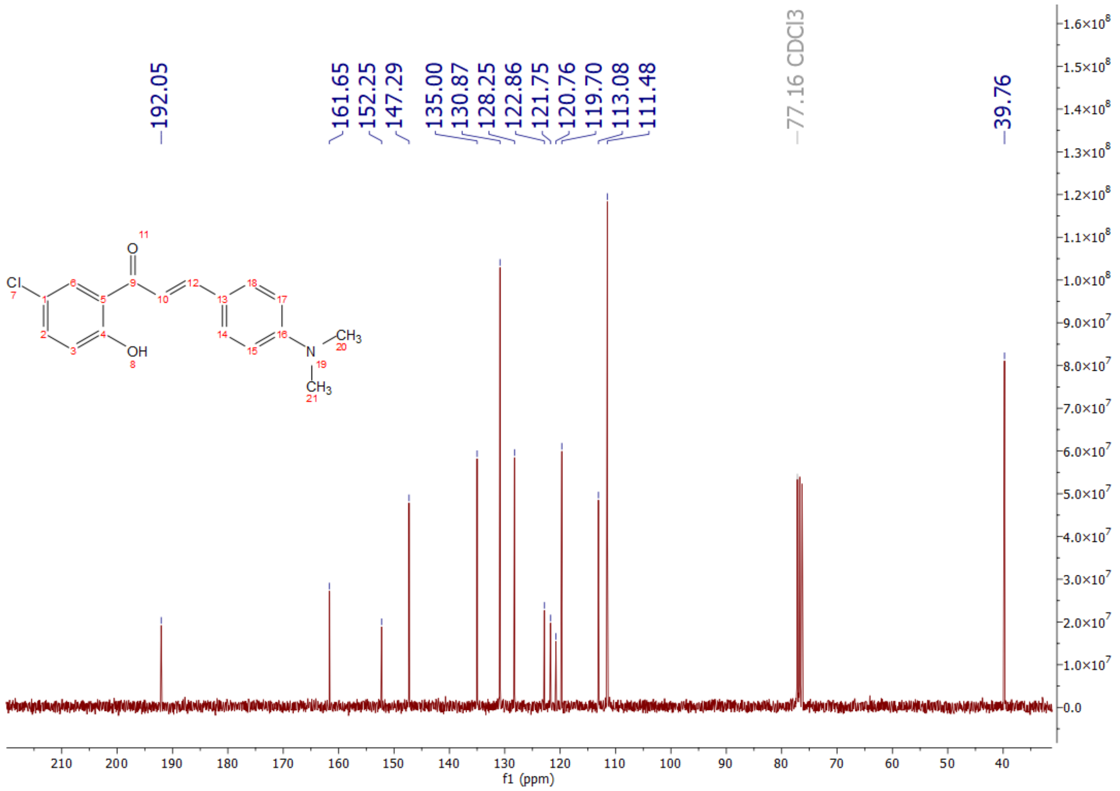

Spectra 18: ^{13}C NMR of compound **3c**

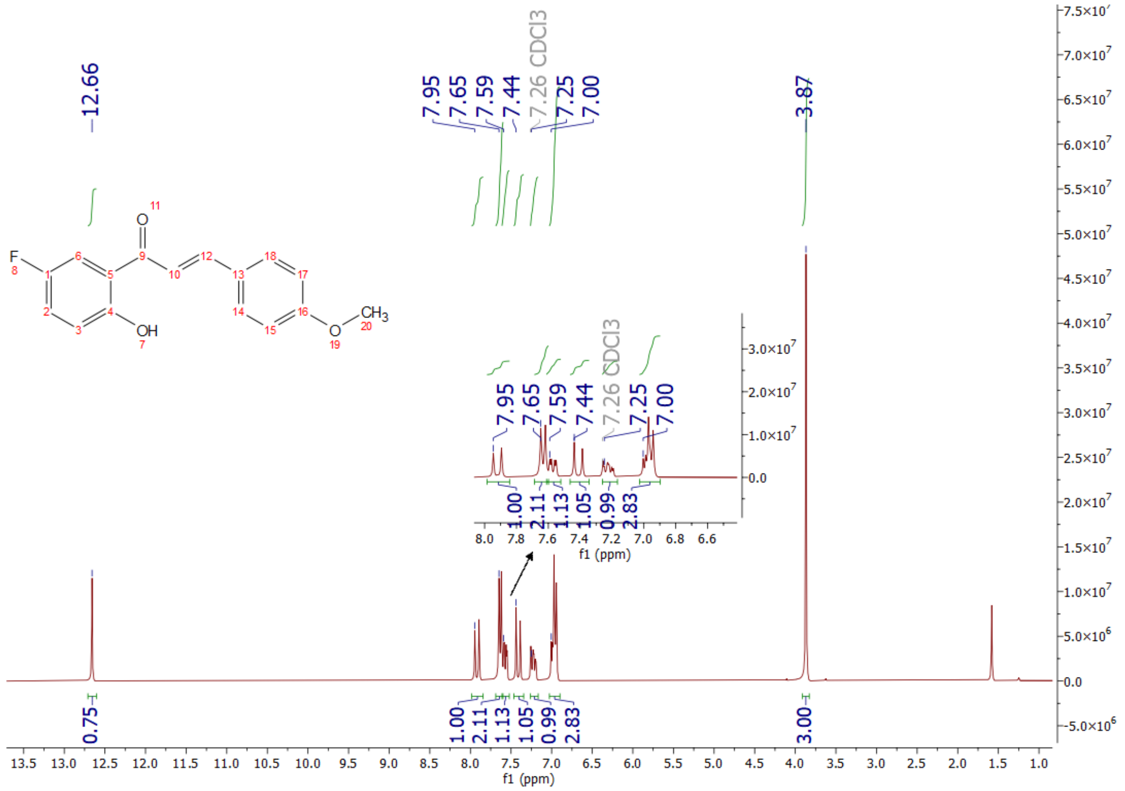

Spectra 19: ^{1}H NMR of compound **4a**

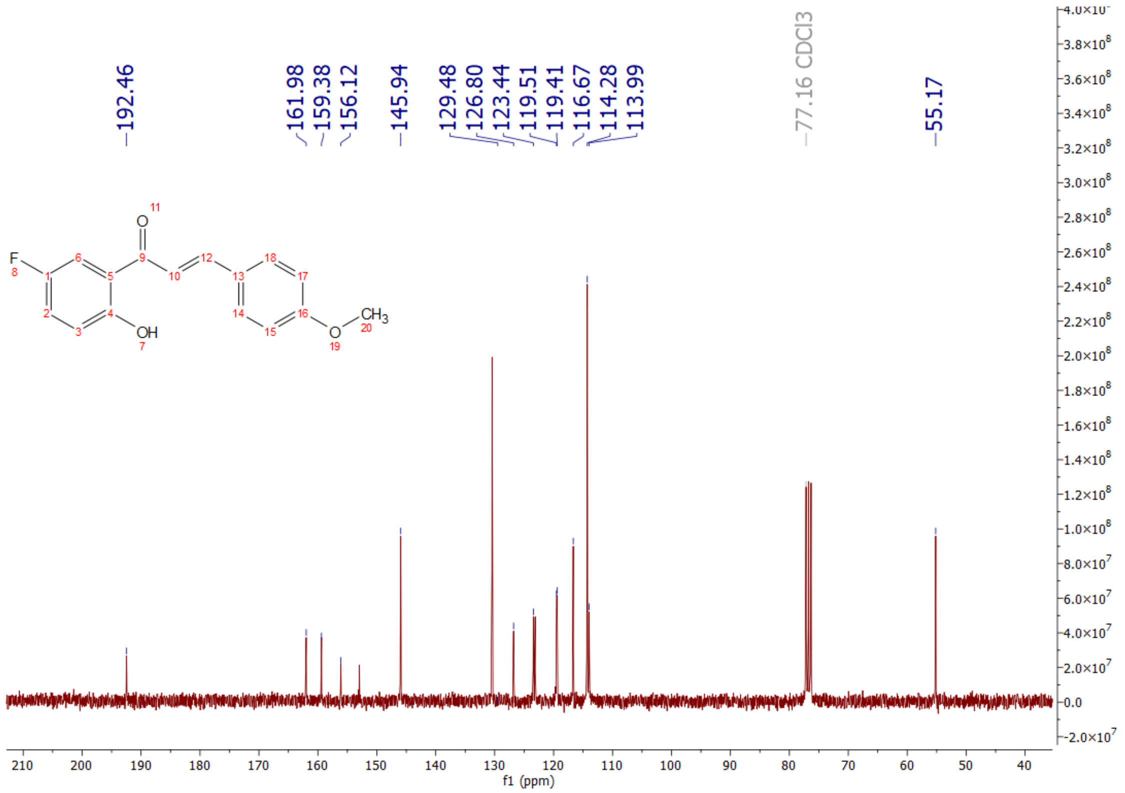

Spectra 20: ^{13}C NMR of compound **4a**

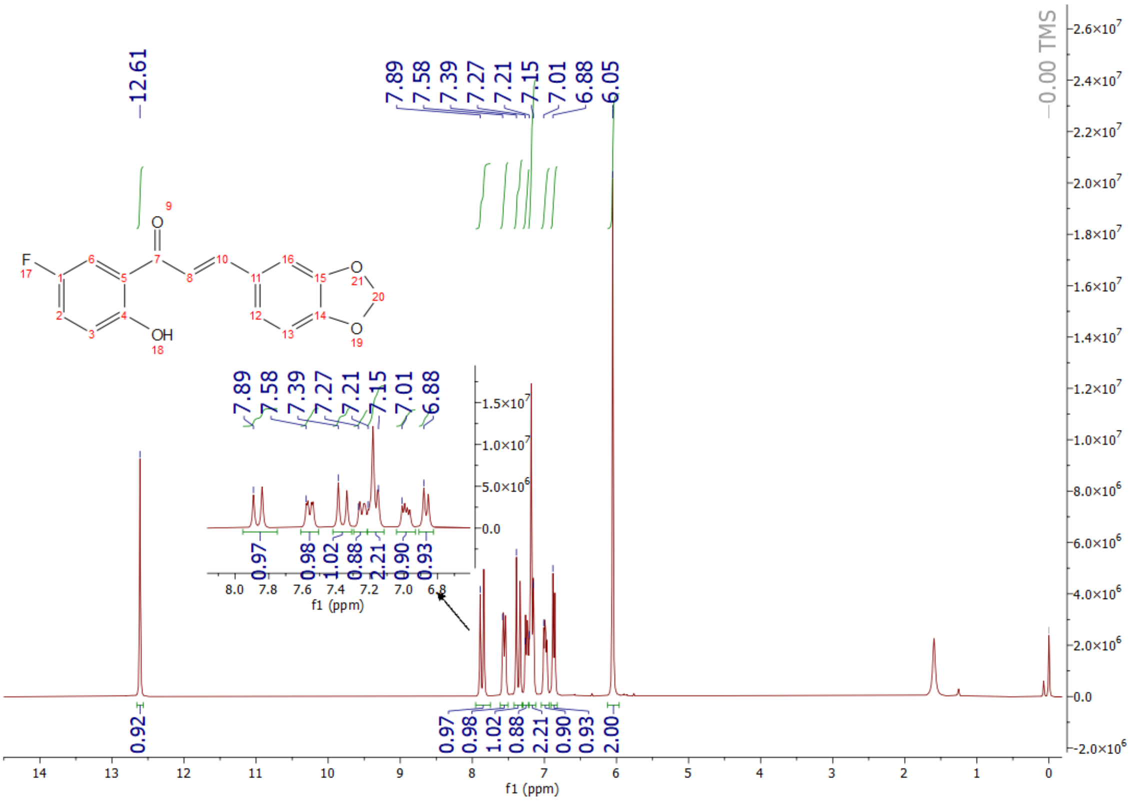

Spectra 21: ^{1}H NMR of compound **4b**

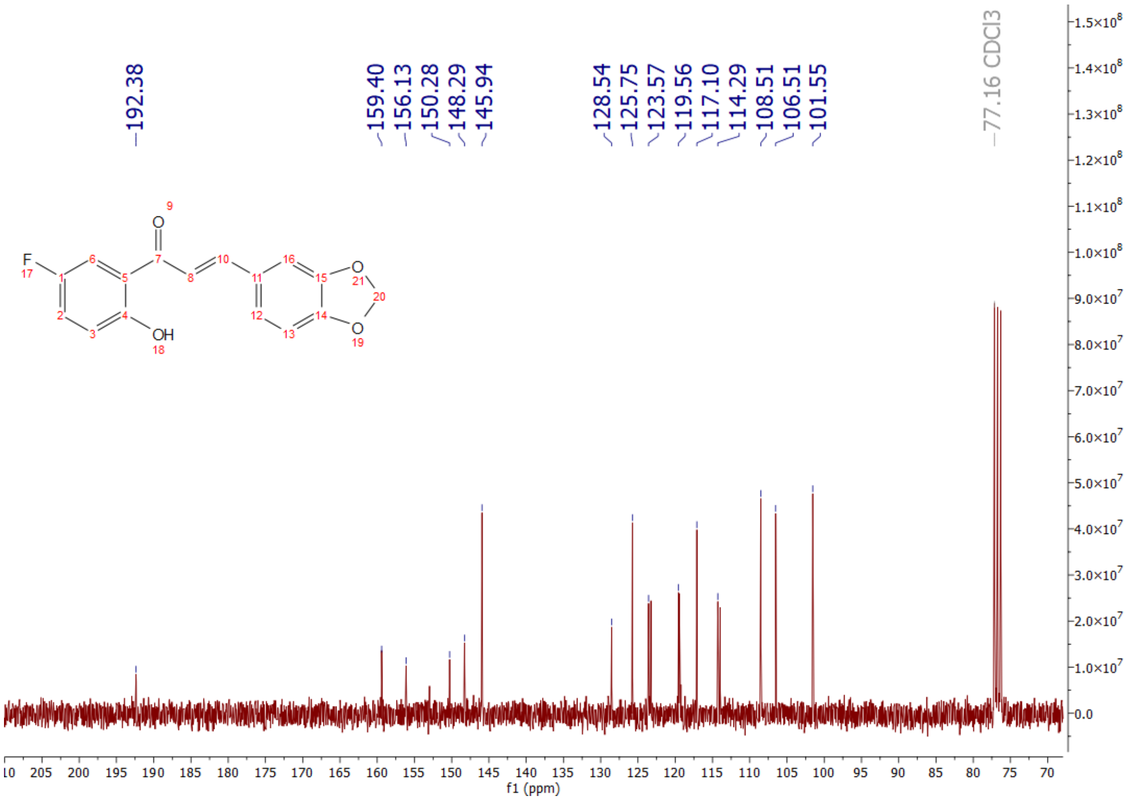

Spectra 22: ^{13}C NMR of compound **4b**

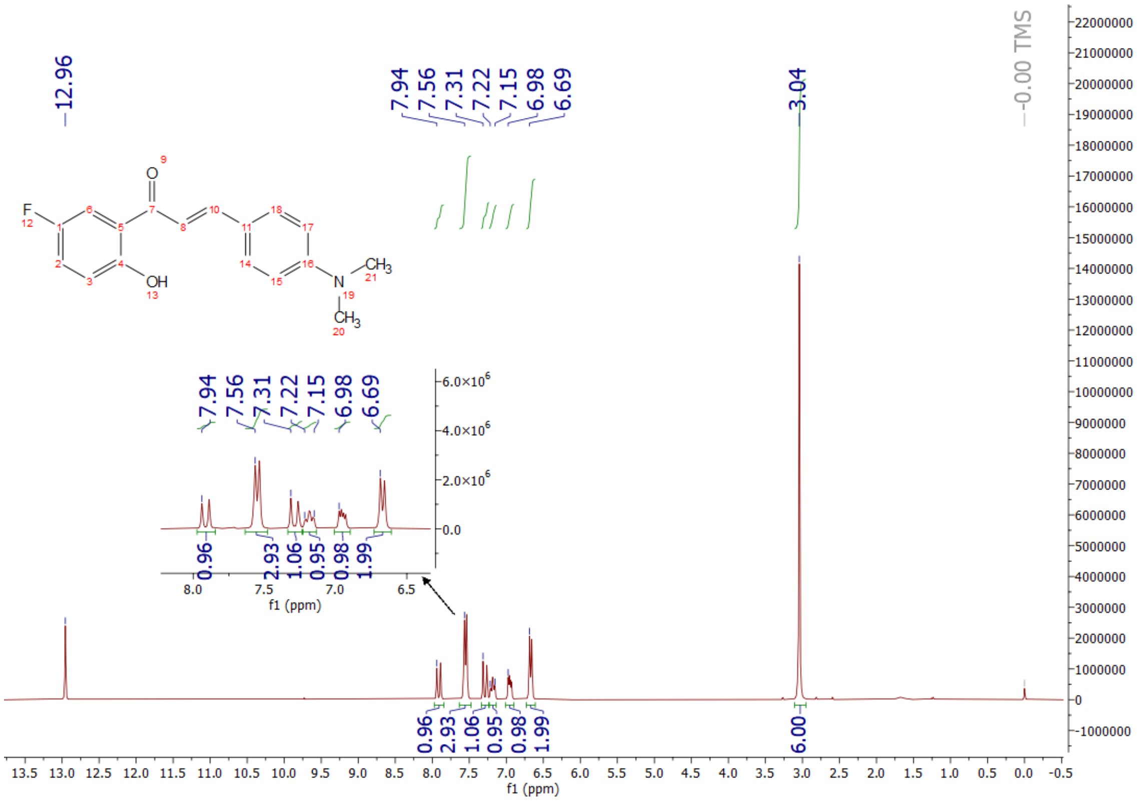

Spectra 23: ^{1}H NMR of compound **4c**

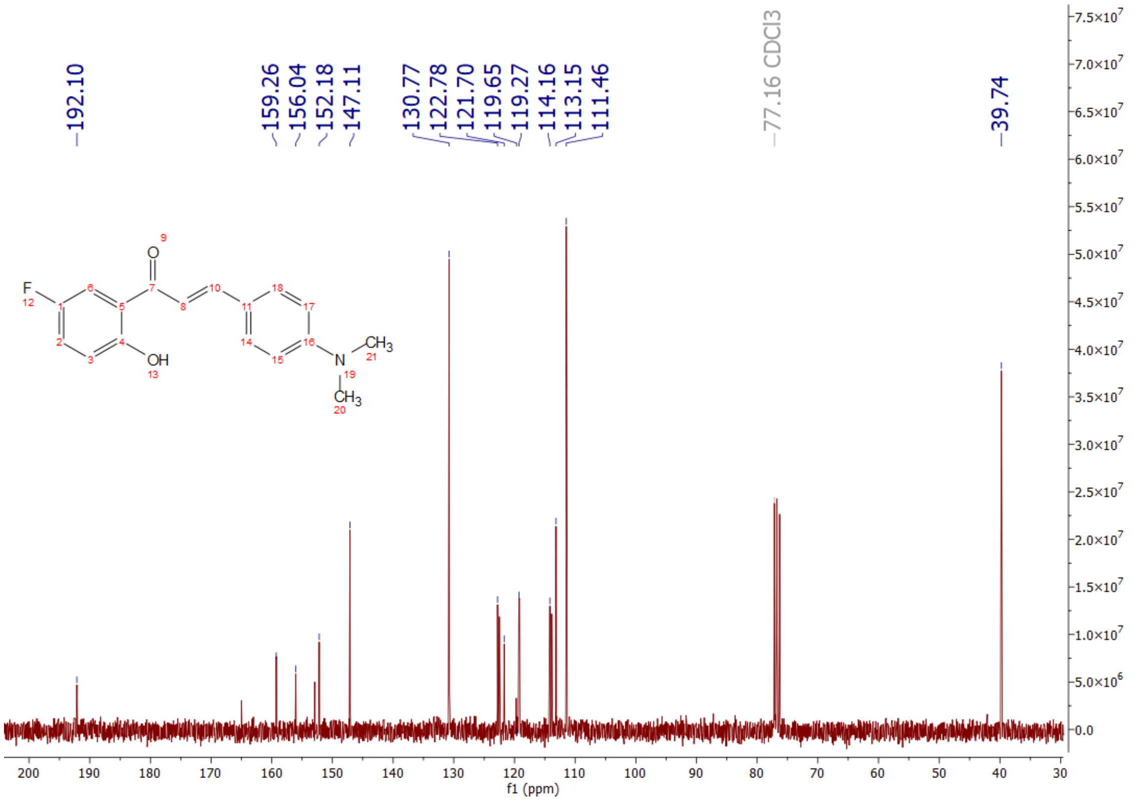

Spectra 24: ^{13}C NMR of compound **4c**

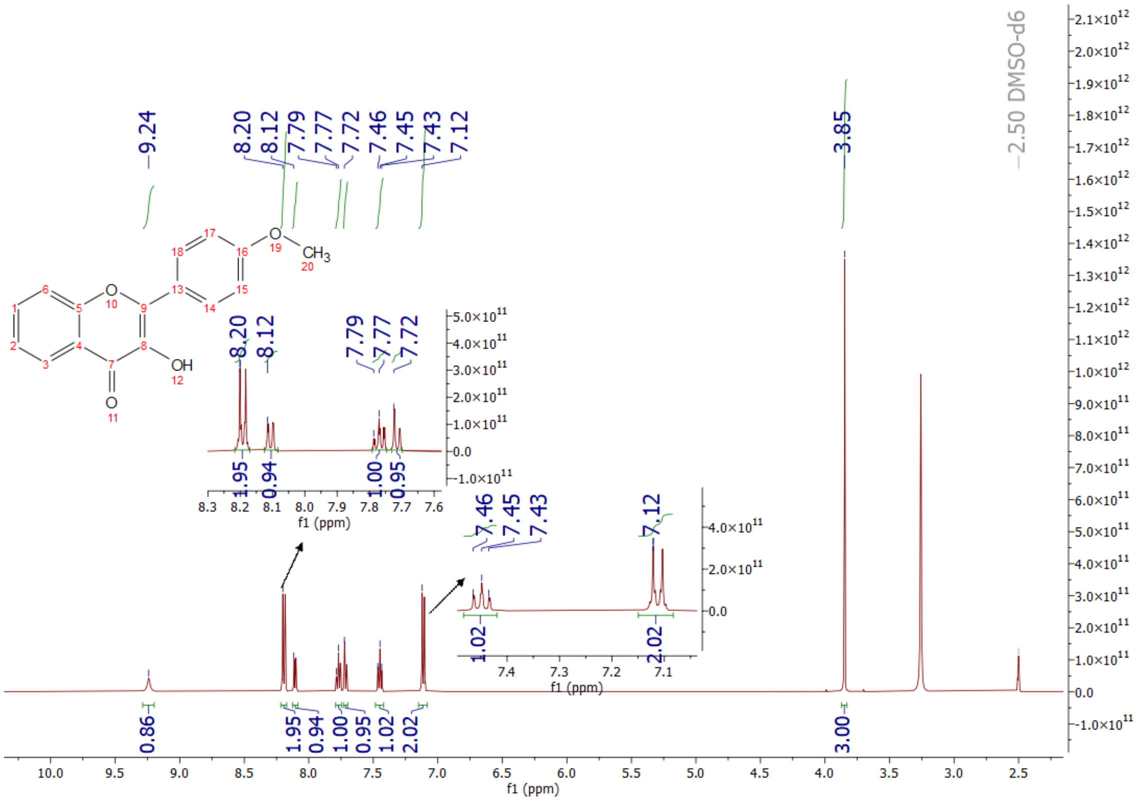

Spectra 25: ^{1}H NMR of compound **f12a**

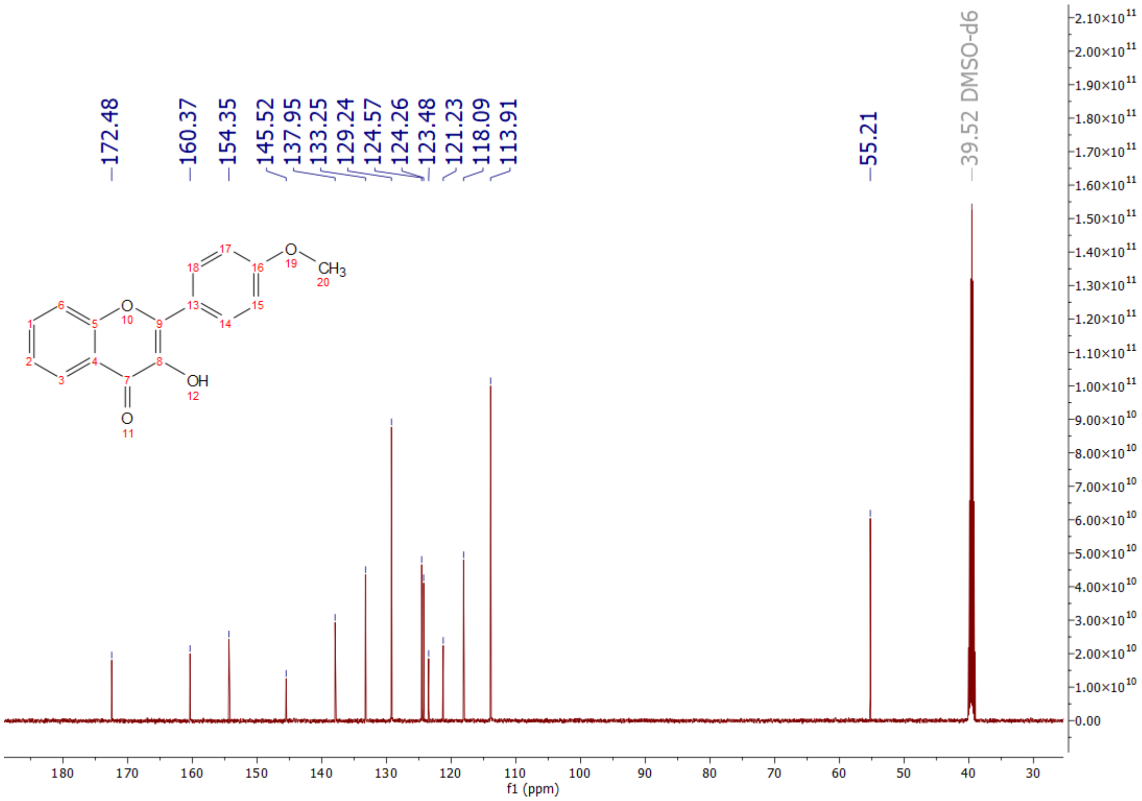

Spectra 26: ^{13}C NMR of compound **f12a**

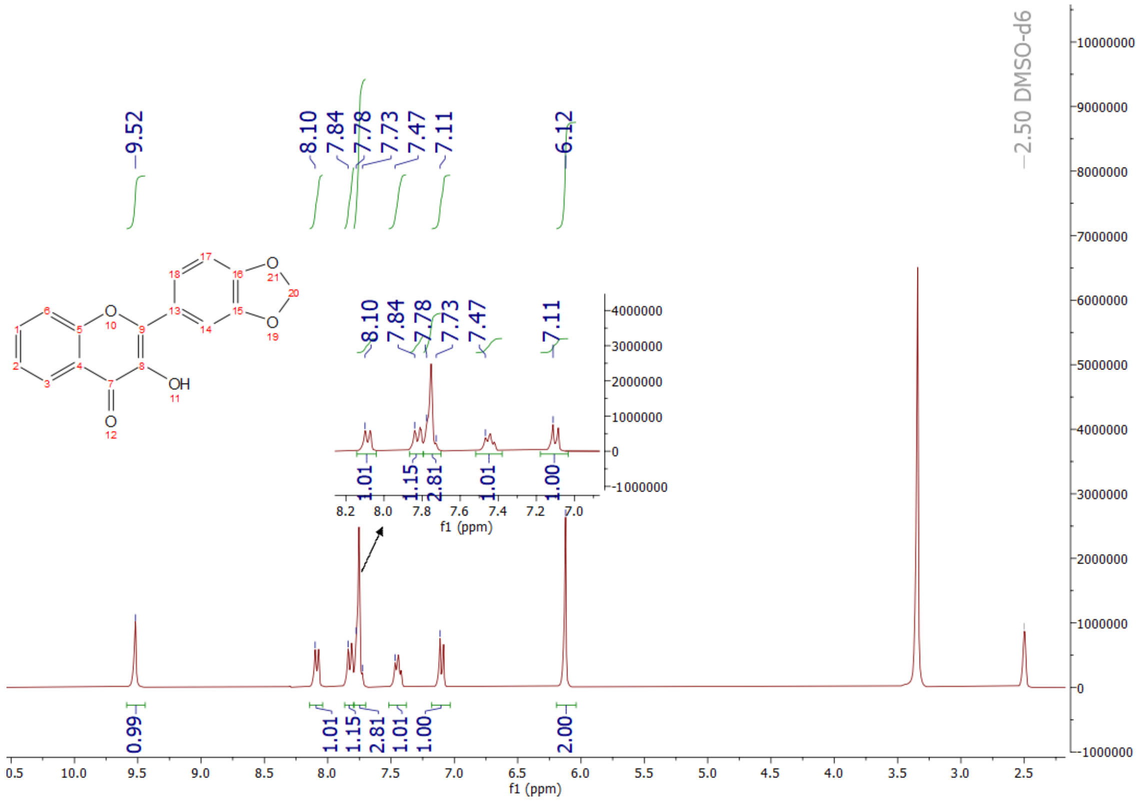

Spectra 27: ^{1}H NMR of compound **f1b**

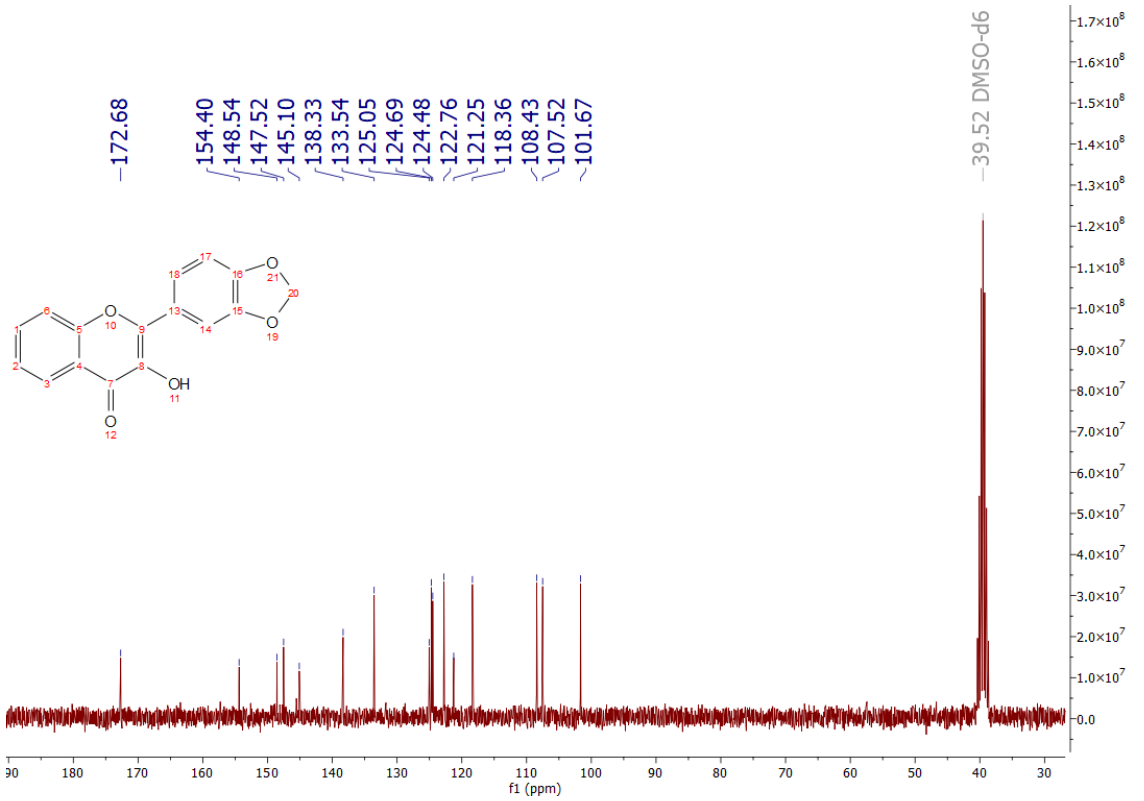

Spectra 28: ^{13}C NMR of compound **f12b**

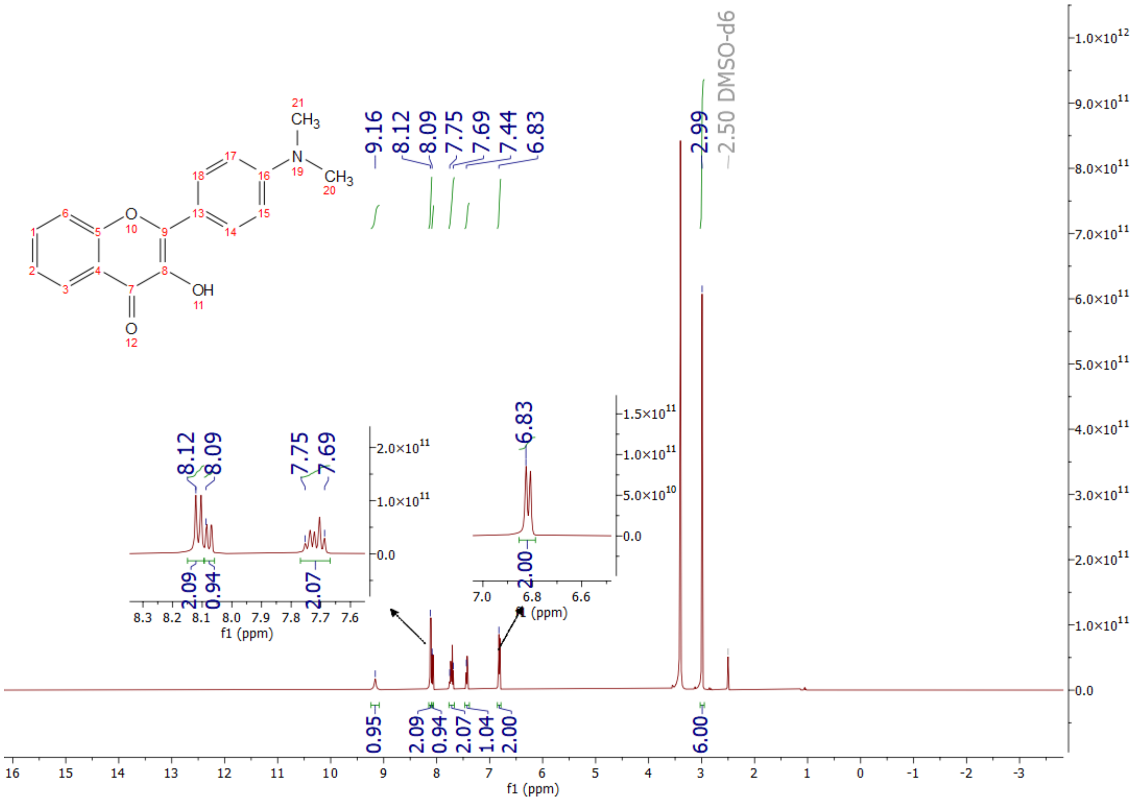

Spectra 29: ^{1}H NMR of compound **f1c**

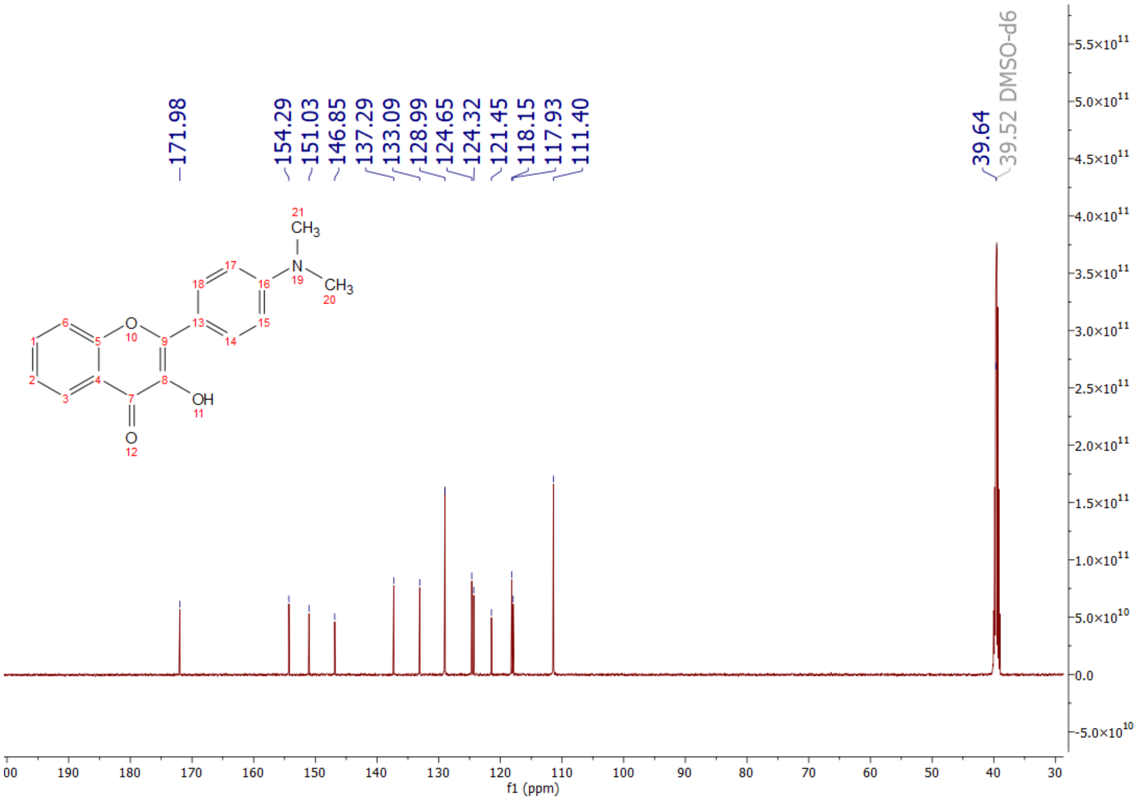

Spectra 30: ^{13}C NMR of compound **f12c**

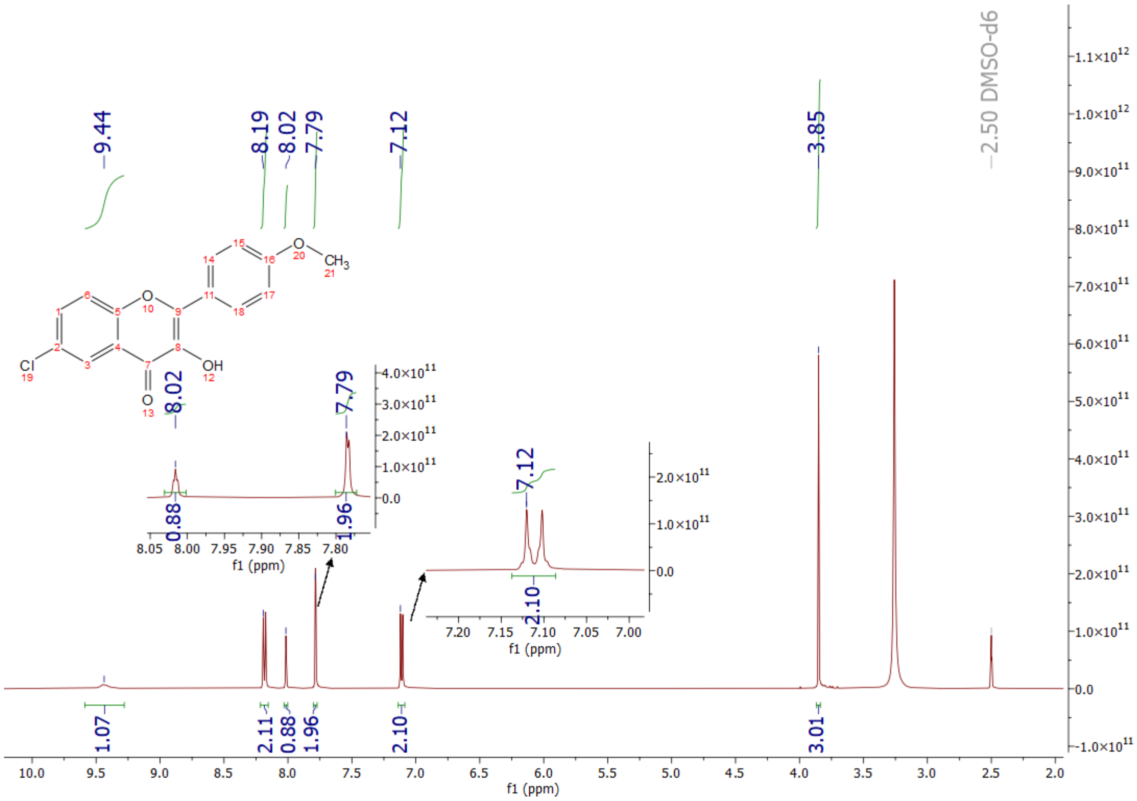

Spectra 31: ^{1}H NMR of compound **f3a**

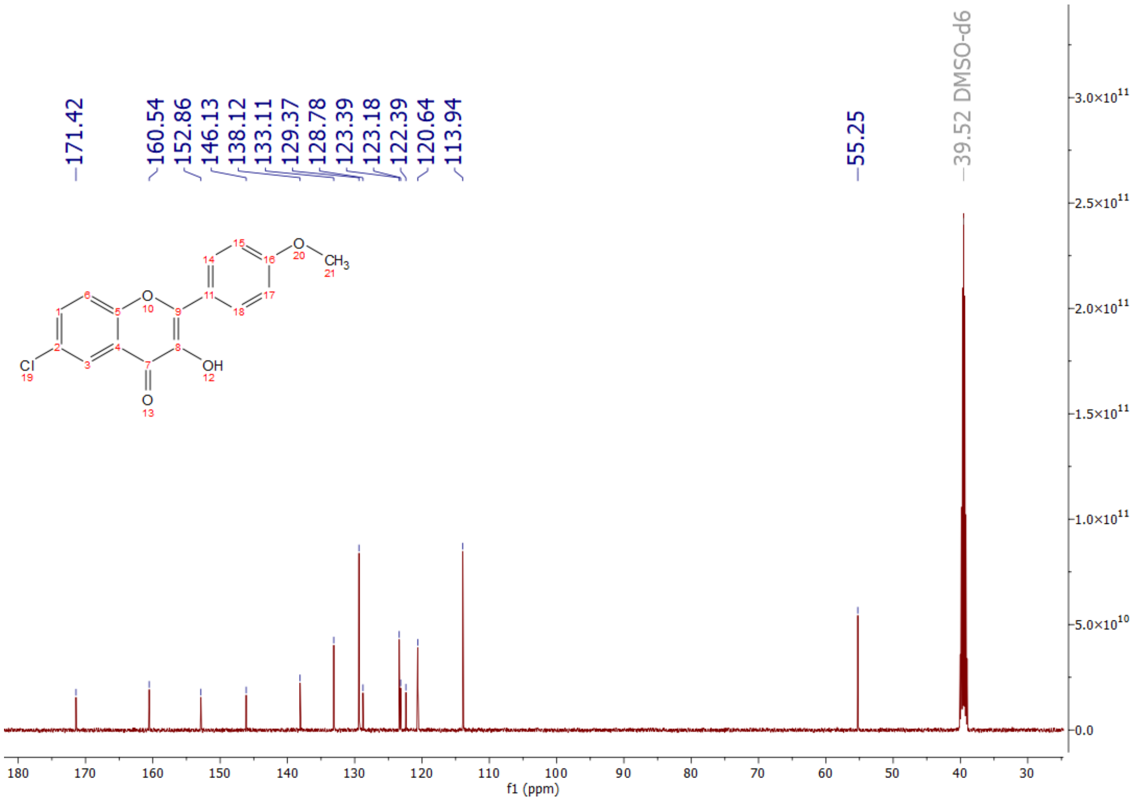

Spectra 32: ^{13}C NMR of compound **f13a**

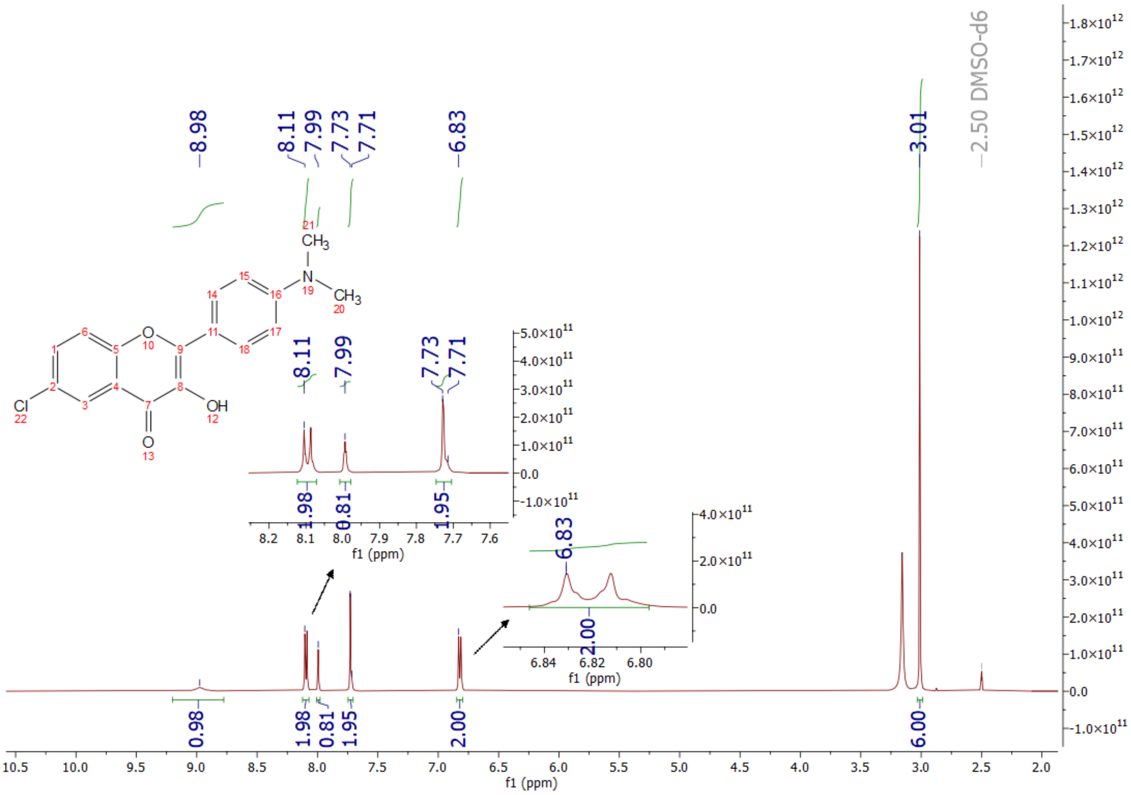

Spectra 33: ^{1}H NMR of compound **f3c**

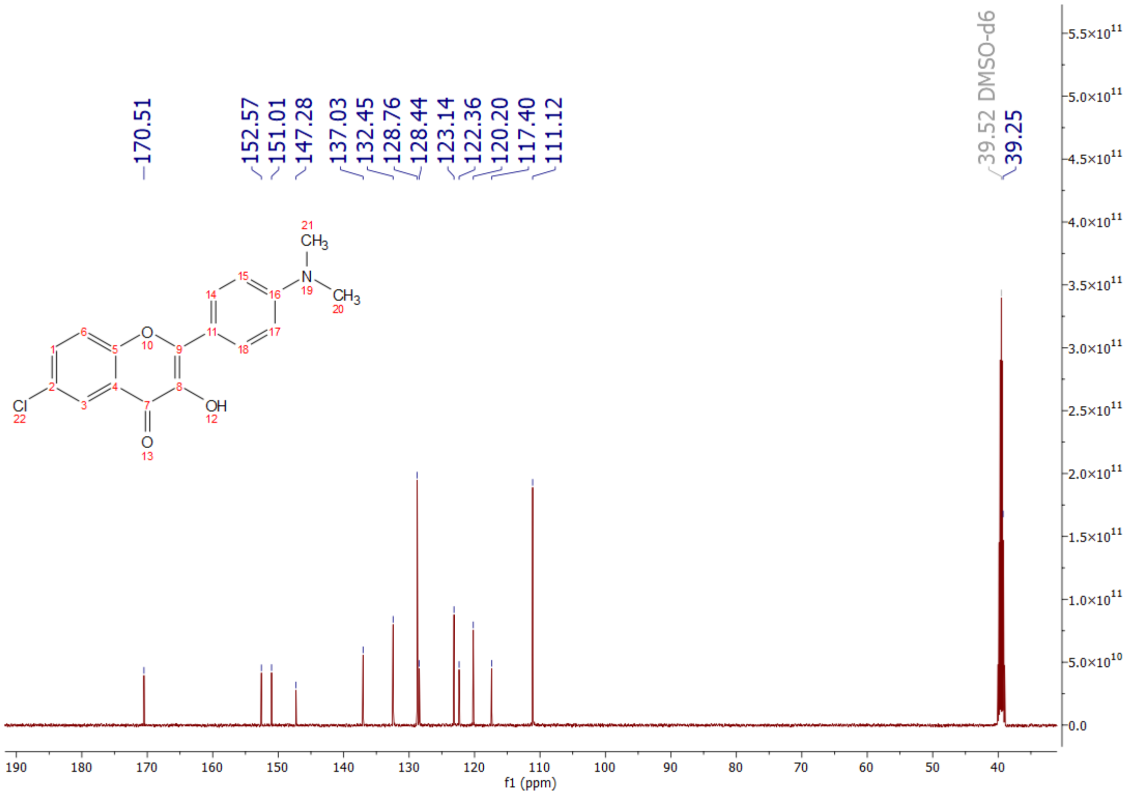

Spectra 34: ^{13}C NMR of compound **f13c**
